# Determining the Effects of Differential Expression of GRKs and β-arrestins on CLR-RAMP Agonist Bias

**DOI:** 10.3389/fphys.2022.840763

**Published:** 2022-03-29

**Authors:** Abigail Pearce, Theo Redfern-Nichols, Matthew Harris, David R. Poyner, Mark Wigglesworth, Graham Ladds

**Affiliations:** ^1^ Department of Pharmacology, University of Cambridge, Cambridge, United Kingdom; ^2^ School of Life and Health Sciences, Aston University, Birmingham, United Kingdom; ^3^ Hit Discovery, Discovery Sciences, BioPharmaceuticals R&D, AstraZeneca, London, United Kingdom

**Keywords:** GPCRs (G protein-coupled receptors), signalling bias, CLR, β-arrestins, RAMPs, internalisation, GRK (G protein receptor kinase)

## Abstract

Signalling of the calcitonin-like receptor (CLR) is multifaceted, due to its interaction with receptor activity modifying proteins (RAMPs), and three endogenous peptide agonists. Previous studies have focused on the bias of G protein signalling mediated by the receptor and receptor internalisation of the CLR-RAMP complex has been assumed to follow the same pattern as other Class B1 G Protein-Coupled Receptors (GPCRs). Here we sought to measure desensitisation of the three CLR-RAMP complexes in response to the three peptide agonists, through the measurement of β-arrestin recruitment and internalisation. We then delved further into the mechanism of desensitisation through modulation of β-arrestin activity and the expression of GPCR kinases (GRKs), a key component of homologous GPCR desensitisation. First, we have shown that CLR-RAMP1 is capable of potently recruiting β-arrestin1 and 2, subsequently undergoing rapid endocytosis, and that CLR-RAMP2 and -RAMP3 also utilise these pathways, although to a lesser extent. Following this we have shown that agonist-dependent internalisation of CLR is β-arrestin dependent, but not required for full agonism. Overexpression of GRK2-6 was then found to decrease receptor signalling, due to an agonist-independent reduction in surface expression of the CLR-RAMP complex. These results represent the first systematic analysis of the importance of β-arrestins and GRKs in CLR-RAMP signal transduction and pave the way for further investigation regarding other Class B1 GPCRs.

## Introduction

Most G protein-coupled receptors (GPCRs) are capable of trafficking to the plasma membrane and signalling in the absence of accessory proteins. The calcitonin like receptor (CLR), however, requires one of three receptor activity modifying proteins (RAMP1-3) for functional membrane expression. Each combination forms a distinct receptor, with a different signalling profile (Weston et al., 2016). RAMP and CLR expression vary across different tissues, creating a diverse profile of signalling from just one GPCR. These CLR-based receptors can respond to three endogenous agonists: calcitonin gene-related peptide (CGRP), adrenomedullin (AM), and adrenomedullin 2 (AM2) (Clark et al., 2021). CLR in complex with RAMP1 generates the CGRP receptor (CGRPR), as CGRP, an abundant neuropeptide that also plays roles in the cardiovascular system, is the most potent agonist for this receptor in generating cAMP. CLR-RAMP2 generates the adrenomedullin 1 receptor (AM1R), with AM (a potent vasodilator) being the most potent at this receptor, and CLR-RAMP3 produces the AM receptor 2 (AM2R), where AM and AM2 are equipotent agonists. The cognate receptor for AM2 is unknown but the peptide, analogous to AM and also a vasodilator, is highly expressed in the heart (Clark et al., 2021).

The G protein and downstream signalling bias in response to all three agonists has been well documented for all CLR-RAMP complexes (Weston et al., 2016; Clark et al., 2021). However, the signalling of GPCRs is not limited to membrane G protein signalling. There is significant evidence that GPCRs are able to signal via β-arrestins, originally identified as terminators of G protein signalling (reviewed Gurevich and Gurevich, 2019). Furthermore, GPCRs, including CLR, are able to signal once internalised, from endosomes (Cahill et al., 2017; Yarwood et al., 2017; Nguyen et al., 2019). It is therefore important to consider β-arrestin recruitment and receptor internalisation when investigating the signalling bias of CLR.

Previously, GPCR signalling was thought of as binary, with agonists activating the receptor before being “turned off”; this is now deemed an oversimplification of the process. G protein-coupled receptor kinases (GRKs), which phosphorylate the receptor following prolonged stimulation, enable the recruitment of β-arrestins. This forms the start of the pathway for homologous desensitisation, leading to receptor internalisation via clathrin-coated pits. There are 7 GRKs, with GRK2-6 ubiquitously expressed and GRK1/7 restricted to photoreceptors. The extent to which each promotes β-arrestin recruitment at different receptors varies. Each phosphorylates different serine and threonine residues on the intracellular region of the GPCR (primarily the C terminal tail), leading to the so-called phosphorylation barcode (Nobles et al., 2011). This barcode, together with the recruitment of β-arrestins, can mediate β-arrestin-dependent signalling. However, it is not possible to predict the pattern in which GRKs phosphorylate receptors, as the consensus sequence for these phosphorylation sites is not fully established. Different patterns of phosphorylation are thought to convey different receptor conformational states, correlating with different downstream signalling pathways (Liggett, 2011). Similarly, activation of different G proteins can correlate with receptor phosphorylation by different GRKs; Gαq activity has recently been inversely linked with GRK5/6-mediated phosphorylation of the angiotensin receptor 1 (AT1R) (Kawakami et al., 2022). In addition to their canonical function, GRKs can be activated by GPCRs but subsequently phosphorylate non-GPCR targets, leading to further signalling cascades (Gurevich et al., 2012; reviewed; Gurevich and Gurevich, 2019). Furthermore, some GRKs have been shown to phosphorylate and sequester the β_2_-adrenoceptor in an agonist independent manner, primarily GRK4, 5, and 6 (Ménard et al., 1996; Andresen, 2010).

To add further complexity to the mechanism of receptor desensitisation, there are two β-arrestin proteins, each with different signalling profiles. Following recruitment to the GPCR, β-arrestins were classically thought to sterically hinder the G protein, blocking further signalling mediated by this pathway and promote desensitisation through clathrin-mediated internalisation. However, recently it has become apparent that some GPCRs can continue to signal once internalised from the endosome. It is now appreciated that the GPCR-β-arrestin complex can assume two distinct conformations. The first is where the β-arrestin only binds to the phosphorylated tail of the GPCR, so facilitating internalisation without blocking G protein signalling; a so called GPCR-G protein-β-arrestin megaplex (Thomsen et al., 2016; Cahill et al., 2017; Nguyen et al., 2019). In the second conformation, the β-arrestin adopts a closed conformation binding to the intracellular core of the GPCR, blocking G protein access (Nguyen et al., 2019). Beyond their role in blocking G protein signalling and mediating internalisation, β-arrestins are believed to act as scaffold proteins, eliciting further signalling pathways. These signalling pathways are distinct from those which are G protein-mediated, and in some cases therapeutically favourable. Some orthosteric (Wisler et al., 2007) and allosteric (Slosky et al., 2020) ligands for GPCRs exploit this β-arrestin-biased signalling in order to exert their favourable pharmacological profiles.

The role of each β-arrestin and GRK in GPCR signalling is poorly understood. Some characterisation of the different isoforms’ roles has been performed, primarily on certain class A GPCRs (Palczewski et al., 1995; Fredericks et al., 1996; Nash et al., 2018; Møller et al., 2020). Recently work has looked at the cannabinoid 2 (CB2) receptor, identifying very little effect when expressing each GRK in turn on the signalling capacity of this receptor (Patel et al., 2021). Previous work has shown roles for GRK2, 3, and 4 in increasing CLR-RAMP2 internalisation when stimulated with AM (Kuwasako et al., 2010) and a role for GRK6 in mediating desensitisation of CLR-RAMP1 (Aiyar et al., 2000), but no investigation of the role of GRKs on desensitisation of CLR-RAMP3 has been conducted. We therefore sought to fully characterise the β-arrestin recruitment and internalisation profile of CLR in complex with each of the RAMPs and identify the role of GRKs in the aforementioned.

Here, we examined signalling bias for cAMP, β-arrestin recruitment, and internalisation for each CLR-RAMP complex. Furthermore, we investigated the role of β-arrestins in agonist dependent internalisation, through chemical inhibition with barbadin, and genetic manipulation of β-arrestin expression. The effects of barbadin were inconclusive, with no effect on receptor internalisation, but a significant decrease in cAMP accumulation. We determined that agonist-stimulated internalisation is dependent on β-arrestins, but cAMP accumulation appeared relatively independent of any internalisation. We then looked at increasing GRK expression, in an attempt to increase β-arrestin recruitment. However, we found that increasing GRK expression (in particular GRK4, 5, and 6) led to an agonist-independent decrease in receptor surface expression, likely due to constitutive phosphorylation and internalisation.

## Methods

### Materials

CGRP, AM, and AM2 were purchased from Bachem and dissolved at 1 mM in water with 0.1% BSA *w/v*. Barbadin (Aobious, Gloucestershire, United Kingdom) was dissolved at 10 mM in DMSO.

### Constructs and Sources

CLR containing a direct C-terminal inframe fusion to NanoLuc was generated in pcDNA3.1(−) (pcDNA3.1(−)-CLR-Nluc) using standard molecular cloning techniques by Sabrina Carvalho (University of Cambridge). pcDNA3.1(+)FLAG-RAMPs and pcDNA3.1-HA-CLR have been described previously (Weston et al., 2016; Harris et al., 2021). pcDNA3.1(+)-hGRKs (Patel et al*.*, 2020) were donated by Professor Michelle Glass and Dr David Finlay (University of Otago). β-arrestin1/2-YFP (Mackie et al*.*, 2019) were donated by Professor Kathleen Caron (Chappell Hill, North Carolina). RIT-Venus (Jimenez-Vargas et al., 2018), Rab5a-Venus, Rab7-Venus, and Rab11-Venus (Jimenez-Vargas et al*.,* 2020) were donated by Luke Pattinson (University of Cambridge).

### Transfection and Cell Culture

HEK293T, HEK293, and HEK293Δβ-arrestin1/2 (donated by Dr Asoka Inoue, Tokyo University) cells were grown in DMEM/F12 Glutamax (ThermoScientific) supplemented with 1% antibiotic-antimycotic solution (AA) and 10% Foetal Bovine Serum (FBS) *v/v*. Cells were grown at 37°C with 5% CO_2_ in a humidified incubator. Cells were transfected using polyethylenimine (PEI, Polyscience Inc.), at a 6:1 ratio of PEI:DNA *v/w*, diluted in 150 mM NaCl.

### Measurement of Intracellular cAMP Accumulation

cAMP accumulation was measured in the HEK293 cell lines as previously described (Knight et al., 2016; Weston et al., 2016). Briefly, cells transfected with CLR-Nluc, FLAG-RAMP1/2/3, GRK2/3/4/5/6, and β-arrestin1/2-YFP (or pcDNA3.1(+) substitutes) in a 1:1:4:5 ratio were harvested using Trypsin-EDTA to bring into single cell suspension, before being resuspended in stimulation buffer (SB, phosphate buffered saline containing 0.1% BSA *w/v*). Cells were plated at 500 cells per well of a 384-well optiplate (PerkinElmer) and stimulated with agonist for 30 min cAMP accumulation was detected using the LANCE *ultra* cAMP detection kit on a Mithras LB 940 multimode microplate reader (Berthold Technologies). For experiments looking at changing β-arrestin expression, in HEK293T, HEK293 or HEK293Δβ-arrestin1/2 cells, cells were incubated in stimulation buffer containing 500 μM isobutylmethylxanthine (IBMX). For experiments utilising barbadin, cells were preincubated with 10 μM barbadin, or 1% DMSO *v/v* for 30 min prior to stimulation. Data were normalised to the maximal level of cAMP accumulation from cells stimulated with 100 μM Forskolin (Sigma).

### Quantification of β-arrestin Recruitment to CLR-RAMP Complexes

β-arrestin recruitment assays were performed as previously described (Marti-Solano et al., 2020). Briefly, HEK293T cells were transfected with CLR-Nluc, FLAG-RAMP1/2/3, GRK2/3/4/5/6, and β-arrestin1/2-YFP (or pcDNA3.1(+) substitutes) in a 1:1:4:5 ratio. 24 h later cells were seeded onto 0.01% Poly-L-lysine coated white 96-well CulturPlates (Perkin Elmer) at 50,000 cells per well in growth media. After 24 h, media was removed, and cells washed in PBS. Cells were then incubated in the dark in buffer containing PBS, 0.49 mM MgCl_2_.6H_2_O, 0.9 mM CaCl_2_.2H_2_O, 0.1% BSA *w/v* and 5 μM of coelenterazine-*h* (Nanolight Technology) for 10 min, before addition of agonist in the range 1 μM to 10 pM. β-arrestin recruitment was recorded for at least 20 min, at 60 s intervals, and measured via a change in the BRET ratio between the donor (*λ* = 460 nm) and acceptor (*λ* = 530 nm), using a Mithras LB 940 multimode plate reader.

### Quantification of CLR-RAMP Internalisation and Localisation to Endosomal Compartments

HEK293T, HEK293, or HEK293Δβ-arrestin1/2 cells were transfected with CLR-Nluc, FLAG-RAMP1/2/3, and a Venus tagged membrane GTPase (RIT), or endosomal markers Rab5a, Rab7, or Rab11, at a 1:1:5 ratio. After 24 h, cells were seeded onto 0.01% Poly-L-lysine coated white 96-well CulturPlates in growth media. Following a further 24 h, media was removed, and cells washed with Krebs (125 mM NaCl, 2.5 mM KCl, 25 mM NaHCO_3_, 1.2 mM NaH_2_PO_4_, 1.2 mM MgCl_2_, 2.5 mM CaCl_2_) containing 0.1% BSA w/v before being incubated in 0.1% NanoGlo reagent (Promega) v/v for 5 min. Where barbadin was used, cells were incubated in KREBs buffer containing 10 μΜ inhibitor for 30 min prior to addition of NanoGlo reagent. Agonists were added in the range 1 μM to 0.1 nM and internalisation measured over 60 min at 120 s intervals via a change in the BRET ratio between the donor (*λ* = 460 nm) and acceptor (*λ* = 530 nm), using a Mithras LB 940 multimode plate reader.

### Reverse Transcriptase PCR to Determine Endogenous Expression of GRKs in HEK293 Cells

RNA was extracted from HEK293 and HEK293T cells using RNeasy mini kit (Qiagen) as per the manufacturer’s protocol. Complementary DNA was generated using the QuantiTect reverse transcription kit (Qiagen) following the manufacturer’s instructions with minus Reverse Transcriptase negative controls performed simultaneously. PCR amplification was performed as previously described (Bailey et al., 2019; Safitri et al., 2020) using the following gene-specific primers: GADPH, forward (5′-TGC​ACC​ACC​AAC​TGC​TTA​GC-3′) and reverse (5′-GGC​ATG​GAC​TGT​GGT​CAT​GAG-3′); GRK1, forward (5′-GGA​GTT​TGA​GAG​TGT​GTG​CTT-3′) and reverse (5′-GCT​TCT​CTG​CCG​ATT​GTA​GGA-3′); GRK2, forward (5′-TCC​AGC​CAT​ACA​TCG​AAG​AGA-3′) and reverse (5′-CAA​AAC​CGT​GTG​AAC​TTA​TCG​C-3′); GRK3, forward (5′-CCG​ATG​TCA​GTT​ACC​TGA​TGG​C-3′) and reverse (5′-GCA​GGA​CGA​TCC​TCT​TGC​T-3′); GRK4, forward (5′-GGA​AAG​GCA​ACC​CGT​AAC​AAA-3′) and reverse (5′-AGG​CGC​AAA​CCT​CTC​CAA​ATC-3′); GRK5 forward (5′-CCA​ACA​CGG​TCT​TGC​TGA​AAG-3′) and reverse (5′-TCT​CTG​TCT​ATG​GTC​CTT​CGG-3′); GRK6, forward (5′-GAG​AAC​ATC​GTA​GCG​AAC​ACG-3′) and reverse (5′-CAG​GCT​GTG​ATA​GTC​ACG​CTC-3′); β-arrestin1, forward (5′-AAA​GGG​ACC​CGA​GTG​TTC​AAG-3′) and reverse (5′-CGT​CAC​ATA​GAC​TCT​CCG​CT-3′); β-arrestin2, forward (5′-TCC​ATG​CTC​CGT​CAC​ACT​G-3′) and reverse (5′-ACA​GAA​GGC​TCG​AAT​CTC​AAA​G-3′); RAMP1, forward (5′-CTG​CCA​GGA​GGC​TAA​CTA​CG-3′) and reverse (5′-GAC​CAC​GAT​GAA​GGG​GTA​GA-3′); RAMP2, forward (5′- GGG​GGA​CGG​TGA​AGA​ACT​AT-3′) and reverse (5′-GTT​GGC​AAA​GTG​GAT​CTG​GT-3′); RAMP3, forward (5′-AAC​TTC​TCC​CGT​TGC​TGC​T-3′) and reverse (5′- GAC​GGG​TAT​AAC​GAT​CAG​CG-3′); CLR, forward (5′-ACC​AGG​CCT​TAG​TAG​CCA​CA-3′) and reverse (5′-ACA​AAT​TGG​GCC​ATG​GAT​AA-3′). Products were resolved on a 2% agarose gel and imaged using a G:Box iChemi gel documentation system. Densitometry was performed using GeneTools (Syngene) and data were normalized to GAPDH expression.

### Quantification of Cell Surface Expression

For CLR surface expression, HEK293 or HEK293Δβ-arrestin1/2 cells were transfected with HA-CLR, FLAG-RAMP1/2/3 and GRK2/3/4/5/6 at a 1:1:4 ratio. After 48 h, 300,000 cells were washed twice with FACS buffer (PBS supplemented with 1% BSA and 0.03% sodium azide) before and after incubation with phycoerythrin (PE)-conjugated mouse anti-HA monoclonal antibody (BioLegend, diluted 1:200 in FACS buffer) for 1 h at room temperature in the dark. Samples were analysed using a BD Accuri C6 flow cytometer, Ex. λ 488 nm and Em. λ 585 nm. Data were normalised to the median APC intensity of cells transfected with pcDNA3.1 as 0% and HA-CLR + FLAG-RAMP1+ pcDNA3.1 as 100%. For RAMP cell surface expression in the presence and absence of agonist, HEK293T cells were transfected with either HA-CLR or CLR-Nluc and FLAG-RAMP1/2/3 at a 1:1 ratio. After 48 h, cells were washed and treated with appropriate agonist (CGRP for CLR-RAMP1 and AM for CLR-RAMP2/3) or vehicle for 30 min. Cells were then washed with ice cold PBS, harvested, assayed as described above and kept at 4°C throughout. Data were normalized to the median APC intensity of cells transfected with pcDNA3.1 as 0% and vehicle treated HA-CLR + FLAG-RAMP2 cells as 100%. Percentage internalisation is expressed relative to vehicle treated cells and cells expressing pcDNA3.1 + FLAG-RAMP.

### Data Analysis

Pharmacological data was analysed in GraphPad Prism v9.0 (GraphPad Software, San Diego). Data were fitted using the three-parameter logistic equation to obtain concentration-response curves and estimates for values of Emax and pEC_50_. Emax was constrained to below 100 for cAMP accumulation assays, and to below the highest response observed in β-arrestin and internalisation assays, which corresponds to CGRP at CLR-RAMP1. Statistical differences were analysed using either a one-way ANOVA followed by Dunnett’s *post-hoc*, a Kruskal-Wallis One-Way ANOVA test with Dunn’s post-test was used or a two-way ANOVA followed by a Tukey’s multiple comparisons test (for comparisons amongst more than two groups) as appropriate. Where comparisons are made between two groups a two-tailed Student’s t-test was used. cAMP data was normalised to 100 μM forskolin stimulation. The means of individual experiments were combined to generate the concentration-response curves displayed in the figures. Heatmaps were generated using the pEC_50_ values calculated from concentration-response curves of the mean of the data, or using the Emax values from the same data, normalised to the response of the cognate ligand at a given receptor. Where no response was observed in the absence of GRK, the maximal change in pEC_50_ was assumed, and the normalised Emax value used.

## Results

### Quantifying Agonist-dependent Desensitisation Bias at the Three CLR-RAMP Complexes

**FIGURE 1:**
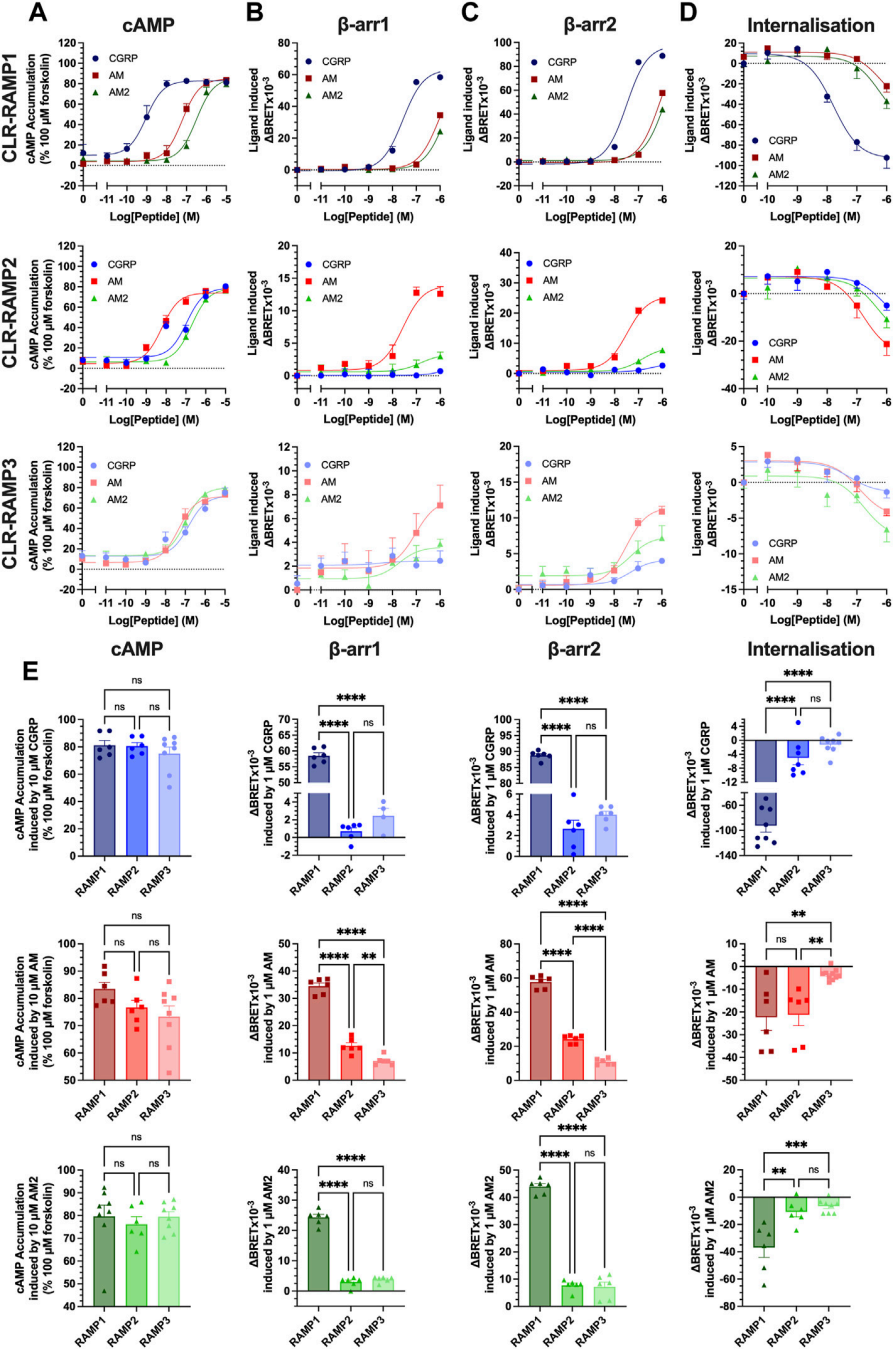
Only cognate agonists of CLR-RAMP complexes recruit β-arrestins and induce internalisation. HEK293T cells expressing CLR-RAMP complexes were assayed for cAMP accumulation **(A)**, β-arrestin1 **(B)**, β-arrestin2 **(C)** recruitment, and internalisation **(D)** at CLR in complex with RAMP1, RAMP2, and RAMP3. **(E)** Responses to 10 μM or 1 μM CGRP, AM, and AM2 at cAMP, β-arrestin1, β-arrestin2, and internalisation. Statistical significance between RAMPs for each peptide was determined, at *p* < 0.05, through One-Way ANOVA with Dunnett’s post-test (*, *p* < 0.05; **, *p* < 0.01; ***, *p* < 0.001; ****, *p* < 0.0001). Data are shown as mean with error bars indicating the SEM of *n* repeats where *n* ranges between 3 and 5 duplicates.

G protein-mediated signalling bias has been well documented for CLR when co-expressed with each of the RAMPs. However, we wanted to examine the bias pattern regarding β-arrestin recruitment and internalisation, as both are important factors in functionality of Class B1 GPCRs, and there is yet to be a global study looking at the desensitisation of each CLR-RAMP complex with all three agonists. We have previously demonstrated HEK293T cells endogenously express low levels of CLR and RAMPs (Bailey et al., 2019), which yields a small response to CGRP and AM (and an even lesser response to AM2), at a far lower potency than would be expected of CLR in complex with any of the three RAMPs (Supplementary Figure S1A). We then determined the suitability of the C-terminal Nluc-tagged CLR with individual FLAG-tagged RAMPs for measuring cAMP accumulation. FLAG-RAMPs have previously been shown to signal comparably to other N terminally tagged RAMPs when coexpressed with CLR (Harris et al., 2021). When compared to HA-CLR, which has previously been used to characterise G protein signalling of the CLR-RAMP complexes (Weston et al., 2016; Harris et al., 2021), CLR-Nluc displayed a reduced potency (∼10-fold compared to HA-CLR), but the same rank order of potency of the three peptides at the different RAMP complexes was observed (Compare Figure 1A with Supplementary Figure S1B).

Given the suitably similar potency profile observed with CLR-Nluc, we next quantified cAMP accumulation, β-arrestin1/2 recruitment (using arrestins each containing an in-frame fusion with a C-terminal YFP) and agonist-dependent internalisation (assayed using a Venus-YFP tagged plasma membrane GTPase RIT) of CLR-Nluc expressed with each FLAG-RAMP (Figures 1A–D; Supplementary Figure S2; Supplementary Table S1). Consistent with our previous reports for cAMP accumulation, CGRP was the most potent agonist at the CGRPR and AM at the AM1R, although all three peptides were observed to be reasonably equipotent at the AM2R. Significantly, these rank orders of potency also translated to both β-arrestins with CGRP being the most potent at RAMP1 (pEC_50_ of 7.56 ± 0.06 at β-arrestin1 and 7.49 ± 0.06 at β-arrestin2), AM at RAMP2 (pEC_50_ of 7.57 ± 0.17 at β-arrestin1 and 7.48 ± 0.09 at β-arrestin2), and all three agonists being equipotent at β-arrestin2 recruitment at RAMP3 (pEC_50_ of 7.31 ± 0.45, 7.49 ± 0.14, and 7.31 ± 0.46 for CGRP, AM, and AM2 respectively). Unlike cAMP accumulation assays, not all agonists were able to elicit β-arrestin recruitment at all CLR-RAMP complexes, with no response observed for CGRP at CLR-RAMP2, and no β-arrestin1 recruitment observed for CGRP at CLR-RAMP3.

In contrast to the cAMP accumulation assays where each CGPR-based peptide was able to act as a full agonist irrespective of the CLR-RAMP complex expressed, only the cognate agonists were able to elicit full β-arrestin recruitment within the concentrations tested. Furthermore, comparison of the overall magnitude of agonist-mediated β-arrestin1/2 recruitment at the respective CLR-RAMP complexes highlighted that all three agonists at CLR-RAMP1 induced significantly higher maximal responses compared to the RAMP2 and RAMP3 complexes (*p* < 0.0001) (Figure 1E). This trend continued in the agonist-induced CLR internalisation assays, suggesting a direct correlation between β-arrestin recruitment and receptor internalisation.

In order to validate the observed internalisation of the three CLR-RAMP complexes, we measured the internalisation of FLAG-RAMP1-3 when co-expressed with HA-CLR or CLR-Nluc. CLR-RAMP complexes are thought to exist in a 1:1 stoichiometry (Hilairet et al., 2001), and internalise as a complex, as such FLAG-RAMP surface expression can be considered a proxy for CLR internalisation. Although FLAG-RAMP1 displayed a higher cell surface expression when co-expressed with HA-CLR than CLR-Nluc, each complex displayed significant internalisation, which was broadly similar across all three RAMPs (Supplementary Figure S3).

### Tracking CLR-RAMP Subcellular Trafficking With Endosomal Markers

**FIGURE 2:**
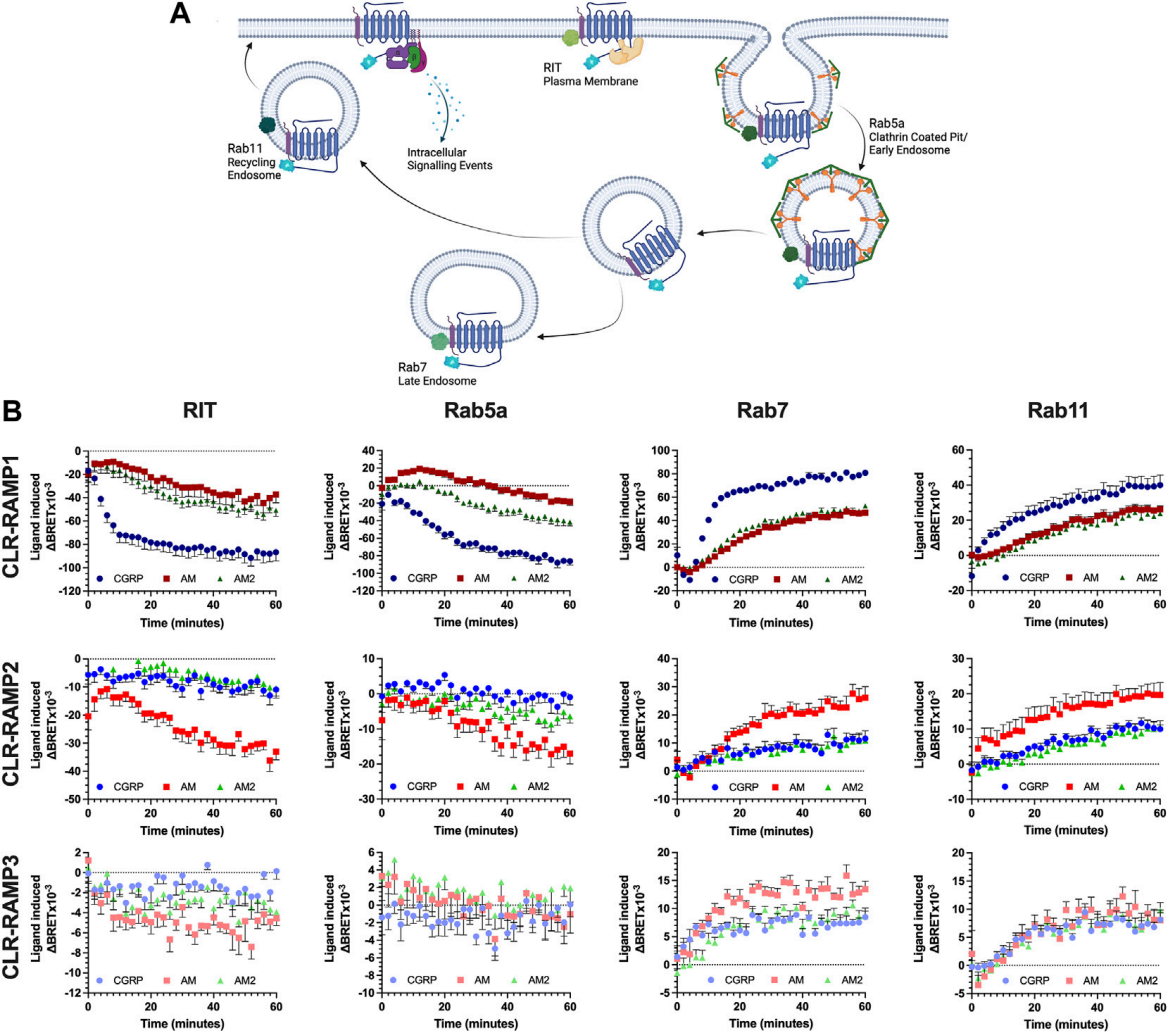
CLR-RAMP1 in the only complex to undergo appreciable internalisation and trafficking, leading towards degradation. Schematic (created with BioRender.com) showing the internalisation and subcellular trafficking of CLR in complex with a RAMP, identifying the different membrane GTPases used **(A)**. Change in colocalisation of CLR over 60 min stimulation with each peptide as determined in HEK293T cells, for RIT, Rab5a, Rab7, and Rab11 when in complex with RAMP1, RAMP2, and RAMP3 **(B)**. Data are shown as mean with error bars indicating the SEM of *n* repeats where *n* = 3 duplicates.

Previous studies have focused on the internalisation and trafficking of a single CLR-RAMP complex. CLR-RAMP1 has been shown to internalise as a complex in a β-arrestin dependent manner (Hilairet et al., 2001; Gingell et al., 2020). Similarly, in response to AM, CLR-RAMP2 underwent internalisation, in a manner dependent on the C terminal tail of the receptor, implicating GRKs and β-arrestins in its internalisation (Kuwasako et al., 2010). The role of the PDZ-interacting domain, found at the C-terminus of RAMP3, on CLR subcellular trafficking has been studied previously; this region is able to interact with subcellular proteins such as the Na+/H+ exchanger regulatory factor-1 (NHERF-1) (Bomberger et al., 2005b) and N-ethylmaleimide-sensitive factor (NSF) (Bomberger et al., 2005a) to regulate internalisation and recycling of the CLR-RAMP3 complex respectively. Comparisons looking at the relative internalisation and trafficking of each CLR-RAMP complex in response to all three peptides have yet to be performed. We utilised RIT as a marker for the plasma membrane, the early endosomal marker Rab5a, as well as the late endosomal marker Rab7 and the recycling endosomal marker Rab11 (Figure 2A), each tagged with Venus-YFP to measure colocalisation with the C-terminal Nluc-tagged CLR.

Consistent with the signalling data, the cognate ligand for each CLR-RAMP complex displayed the greatest internalisation (as quantified by loss of a BRET signal between CLR-Nluc and Venus YFP-RIT) and subcellular trafficking, as determined through colocalization with each GTPase in turn (Figure 2B). This translated across all compartments. Due to the dynamic nature of the passage through the early endosome (rapid entry and departure), only for the cognate agonists at CLR-RAMP1 and -RAMP2 could any substantial change in colocalisation over time be observed. The increase in colocalization with Rab7 suggests CLR is degraded as a means of desensitisation, although colocalization with Rab11 indicates it is capable of recycling back to the membrane. The AM2R (RAMP3-CLR complex) displayed very little change in colocalization with RIT at the plasma membrane or with Rab5a when stimulated with any of the peptides, however some increase in colocalization with Rab7 and Rab11 was observed, indicating a very small level of internalisation does occur.

### Use of the Small Molecular Inhibitor Barbadin Reduces cAMP Accumulation Independently of Internalisation

**FIGURE 3:**
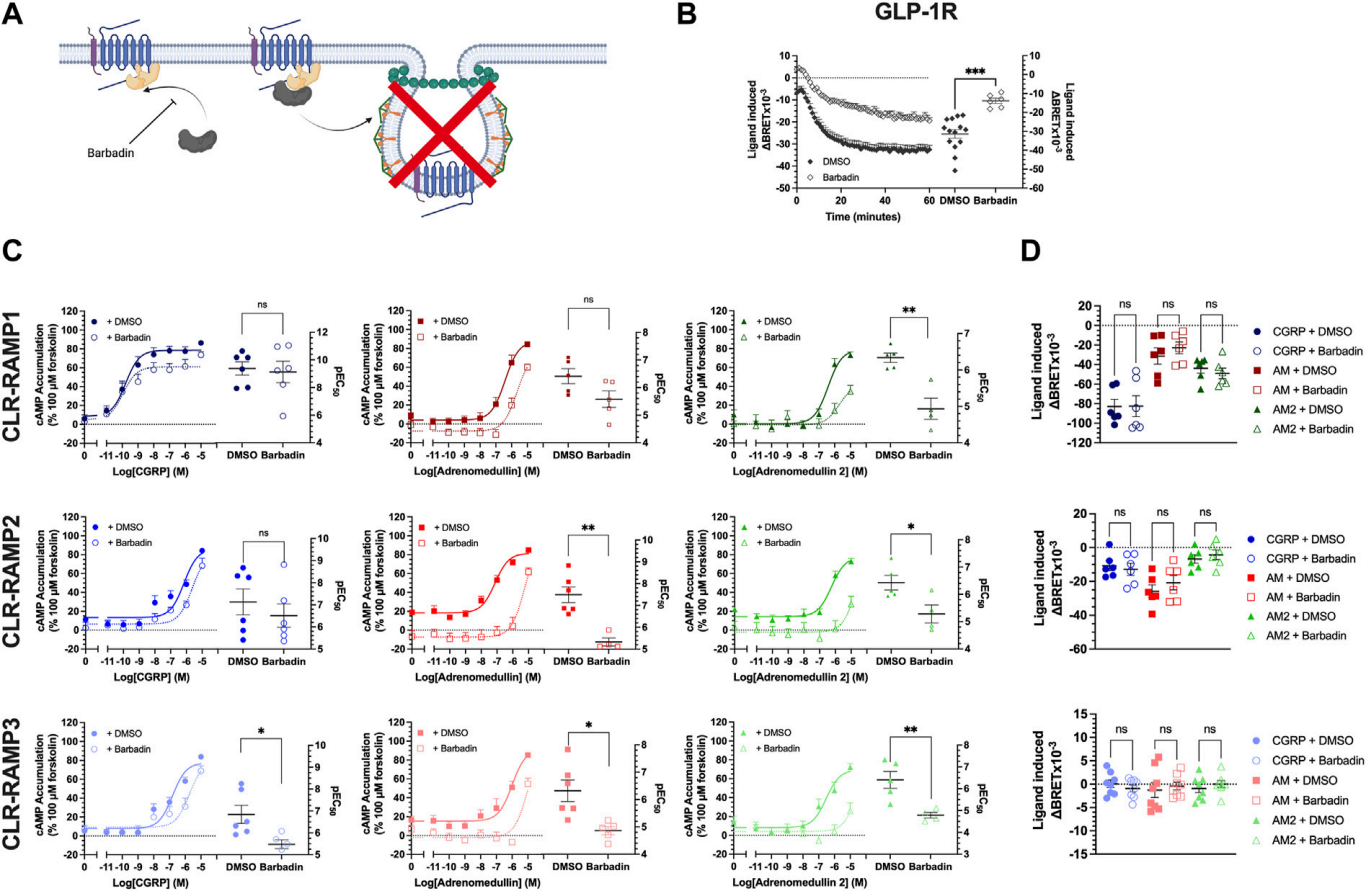
Barbadin significantly impairs cAMP accumulation at CLR-RAMP complexes in an internalisation independent manner. **(A)** Schematic (created with BioRender.com) indicating the role of barbadin, a small molecule inhibitor of the β-arrestin-AP2 complex. **(B)** Effects of barbadin on another Class B1 GPCR, the GLP1R. **(C)** cAMP accumulation mediated by each peptide agonist following a 30 min pretreatment with DMSO (closed symbols, solid line) or 100 μM barbadin (open symbol, dotted line), for RAMP1, RAMP2, and RAMP3. Differences in, and potency values are reported on the adjoining scatter plots. **(D)** Effect of 30 min pretreatment with DMSO (closed symbols) or barbadin (open symbols) on internalisation of CLR-RAMP1, RAMP2, or RAMP3. Statistical significance between vehicle (DMSO) and barbadin treated cells was determined, at *p* < 0.05, using Student’s t-test (*, *p* < 0.05; **, *p* < 0.01). Data are shown as mean with error bars indicating the SEM of *n* repeats where *n* = ranges between 3 and 6 duplicates.

The canonical role of β-arrestins is the desensitisation of G protein signalling; steric hindrance to disrupt G protein-receptor association and acting as scaffolds for proteins which decrease the G protein activity and output e.g. phosphodiesterases (PDEs) (Perry et al., 2002). Additionally, β-arrestins mediate agonist dependent internalisation at many GPCRs, through the recruitment of β2-adaptin (AP2) and subsequently clathrin, to mediate endocytosis. Hence, we attempted to decouple these two mechanisms, using the small molecule inhibitor barbadin. Barbadin is an inhibitor of the β-arrestin-AP2 interacting domain, therefore inhibiting clathrin-mediated endocytosis without inhibiting β-arrestin recruitment (Beautrait et al., 2017) (Figure 3A). In order to validate barbadin, we measured its effects on internalisation of the Glucagon-like peptide-1 receptor (GLP-1R), another Class B1 GPCR which undergoes clathrin-mediated endocytosis following β-arrestin recruitment (Fletcher et al., 2018). A 30 min preincubation with 100 μM barbadin was found to inhibit GLP-1R internalisation (Figure 3), therefore showing it is active at this concentration (Figure 3B).

Barbadin appeared to reduce cAMP accumulation for all agonists at the three CLR-RAMP complexes, although in some instances (e.g. for CGRP at the CLR-RAMP1 complex) these effects did not reach significance (Figure 3C, Supplementary Table S3). At the CLR-RAMP1 complex, the biggest differences were observed when AM2 was used as the stimulating agonist, with barbadin inhibiting signalling, and reducing both the potency and maximal response (pEC_50_ from 6.34 ± 0.13 to 4.93 ± 0.29; *p* = 0.0021, Emax from 79.8 ± 4.5 to 47.2 ± 8.9; *p* = 0.02). When looking at CLR-RAMP2, although the response to CGRP was again unaltered, the response to AM was significantly impaired (pEC_50_ reduced from 7.49 ± 0.36 to 5.33 ± 0.18; *p* = 0.0017). Again, the response to AM2 was far smaller when pretreated with barbadin. At CLR-RAMP3, all three agonists showed reduced potencies at stimulating cAMP accumulation when treated with barbadin (Figure 3C; Supplementary Table S3).

Finally, we then investigated the actions of barbadin treatment of CLR-RAMP complex internalisation (Figure 3D, Supplementary Table S4). Surprisingly, and in contrast to the actions observed at cAMP accumulation, barbadin treatment did not block agonist-mediated CLR internalisation for any CLR-RAMP complex when used at the concentration that blocked GLP-1R internalisation (Figure 3B). This data would suggest that barbadin could not be used to decouple CLR-RAMP internalisation from β-arrestin recruitment.

### CLR Requires β-arrestins to Internalise, and This Internalisation Is Important for the Receptor to Achieve Maximal Signalling

**FIGURE 4:**
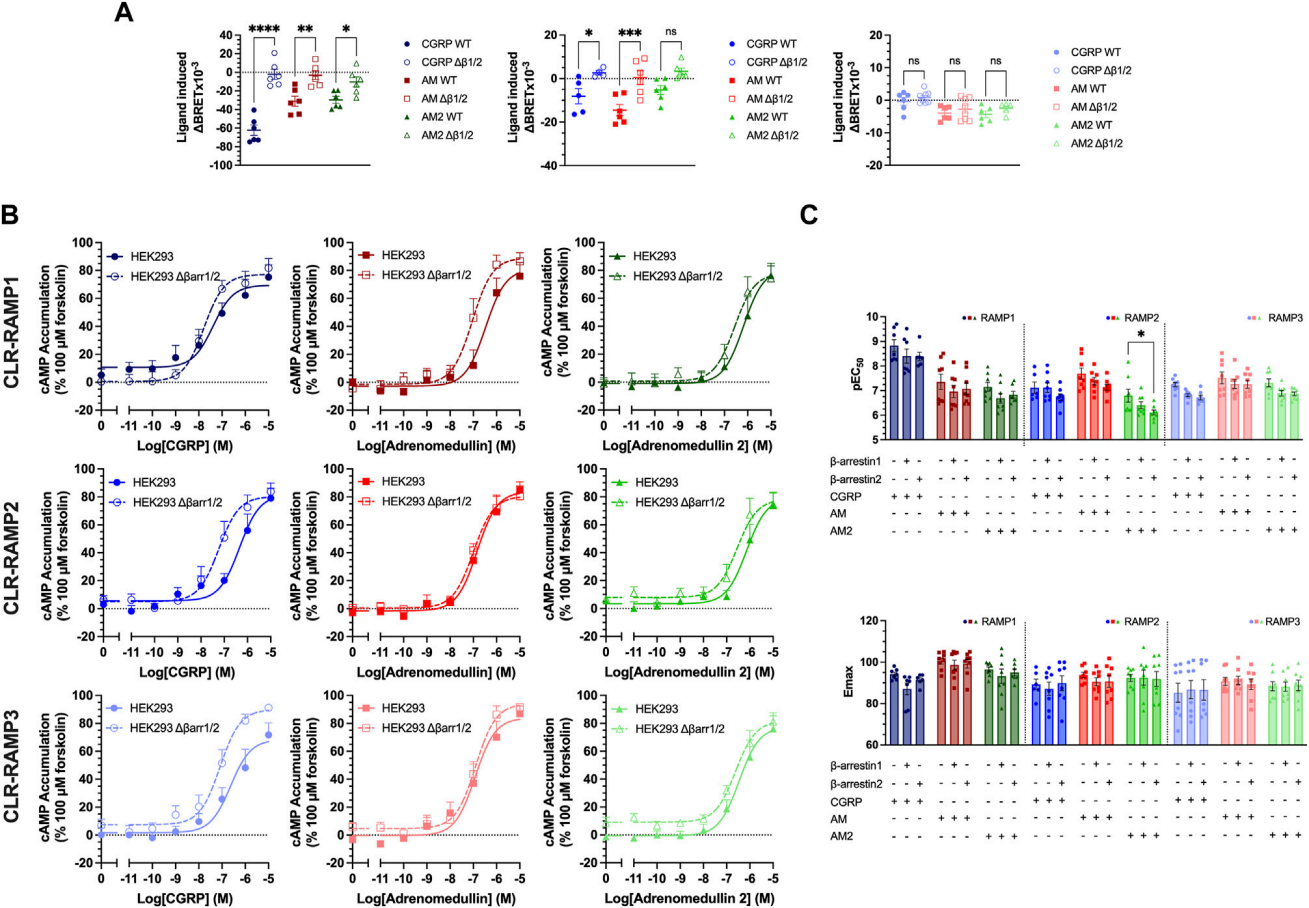
Expression of β-arrestins reduced potency and efficacy of cAMP signalling for all CLR-RAMP complexes. **(A)** Changes in CLR internalisation in complex with RAMP1, RAMP2, or RAMP3 in HEK293 cells expressing (closed symbols) or lacking (open symbols) β-arrestin1/2. Statistical significance between responses in WT and β-arrestin1/2 KO cells was determined, at *p* < 0.05, through Student’s t-test (*, *p* < 0.05; **, *p* < 0.01; ***, *p* < 0.001; ****, *p* < 0.0001). **(B)** Effect of knocking out β-arrestin1/2 on cAMP in cells expressing CLR-RAMP1, RAMP2, or RAMP3. **(C)** Bar charts showing the potency and maximal response of the peptides at CLR with each RAMP in turn, in cells overexpressing β-arrestin1 or 2. All data are mean ± SEM of *n* repeats where *n* ranges between 3 and 4 duplicates. Statistical significance of responses in the presence of overexpressed β-arrestins was compared to the response in the absence of β-arrestin, at *p* < 0.05, using a Two-Way ANOVA.

Due to the inconclusive nature of the effects of barbadin, we then looked at the signalling in cells genetically modified to express different levels of β-arrestins. We used HEK293 cells and a modified line devoid of β-arrestin1 or 2 (O’Hayre et al., 2017), which displayed a similar expression of CLR and RAMPs as HEK293T cells (Supplementary Figure S4). No agonist-induced internalisation was observed in this cell line, leading us to conclude that CLR-RAMP internalisation is β-arrestin-dependent, despite observing no effect of barbadin (Figure 4A, Supplementary Figure S5A, Supplementary Table S5). We repeated the cAMP accumulation experiments in the presence of barbadin in these β-arrestin KO HEK293 cells, and an agonist dependent reduction in cAMP accumulation was again observed (Supplementary Figure S5B), suggesting at least part of the effects of barbadin occur independently of the β-arrestin.

Having considered the effects of deleting β-arrestins on CLR-RAMP internalisation, we next considered their impact on agonist-dependent cAMP accumulation. In HEK293 cells with β-arrestin knocked out, there was a significant trend towards increasing the potency when β-arrestins were knocked out (*p* < 0.0001) (Figure 4B, Supplementary Table S6). This was the greatest for CGRP at CLR-RAMP2 and CLR-RAMP3 (RAMP2, *p* = 0.002, RAMP3, *p* = 0.007). Finally, we determined the effects of overexpression of either β-arrestins on cAMP accumulation from the three CLR-RAMP complexes (Figure 4C Supplementary Figure S5C, Supplementary Table S7). Overall, there was a significant trend towards β-arrestin overexpression decreasing the potency of cAMP accumulation, indicating a small increase in desensitisation (*p* < 0.0001). Whilst this was not significant in most individual cases, there was a significant decrease in potency observed for AM2 at CLR-RAMP2 when β-arrestin2 was overexpressed (*p* = 0.02). It is likely that these effects are only small as HEK293T cells endogenously express high levels of β-arrestin1/2 (Supplementary Figure S6A). Overall, our data is supportive of the notion that RAMP-CLR complexes require β-arrestins to undergo receptor internalisation, and that modulation of β-arrestin expression can influence the potency and magnitude of the signalling response when stimulated with the CGRP-family of peptide agonists.

### Overexpression of GRKs Induces Agonist-independent Internalisation of CLR-RAMP Complexes

**FIGURE 5:**
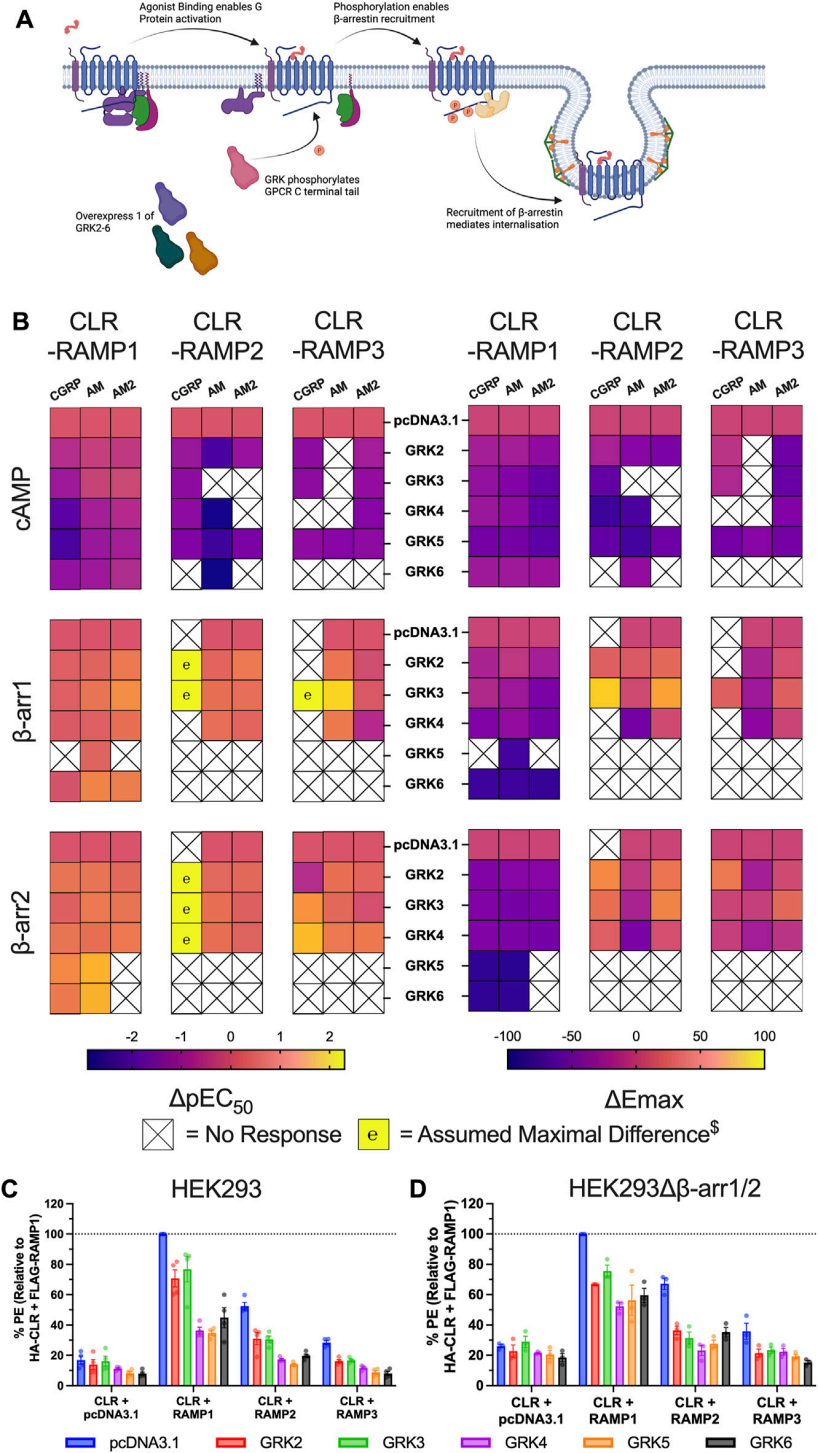
GRK overexpression impairs CLR-RAMP complex signalling through constitutive receptor internalisation. **(A)** Schematic (created with BioRender.com) showing the role of GRKs in mediating GPCR desensitisation and internalisation, and highlighting how we manipulated this system for the experiment. **(B)** Heat maps illustrating the effects of overexpression of each GRK on the potency (left) and Emax (right) on cAMP accumulation, and β-arrestin1/2 recruitment at the three CLR-RAMP complexes when stimulated with CGRP, AM and AM2. $ Maximal change in pEC_50_ was assumed where no response was observed in the absence of GRK. **(C–D)** Cell surface expression of HA-CLR, measured using a phycoerythrin (PE)-conjugated anti-HA antibody, following overexpression of GRKs prior to addition of agonist in HEK293 cells **(C)** and HEK293Δβ-arrestin1/2 cells **(D)**. All data are mean ± SEM of *n* repeats where *n* ranges between 3 and 4 experiments.

There is growing evidence to suggest that different GRKs are responsible for mediating different levels of β-arrestin recruitment. Thus, having established that β-arrestin expression is important for CLR-RAMP complex signalling, we sought to determine which GRKs may be responsible for mediating these effects (Figure 5A). Analysis of GRK expression, using semi-quantitative rt-PCR, indicated that GRK2/3/4/5/6 were all expressed in all HEK293 cell lines in the study (Supplementary Figure S6B). Since both GRK1 and GRK7 expression is restricted to the retina neither was included in our analysis, nor were they included in the functional studies. We then strove to determine the effects of GRK overexpression on cAMP accumulation and β-arrestin recruitment at each CLR-RAMP complex with all three agonists (Supplementary Figures S7–S9). Strikingly, and somewhat surprisingly, overexpression of many of the GRKs appeared to significantly attenuate cAMP accumulation and recruitment of β-arrestins to the CLR-RAMP complexes upon agonist stimulation (Figure 5B, Supplementary Table S8–S10). Of the three CLR-RAMP complexes, CLR-RAMP1 appeared most resistant to GRK-mediated attenuation of signalling. The negative effects of GRK expression were most pronounced for GRK5 and GRK6 at all three CLR-RAMP complexes, with recruitment of β-arrestins to the CLR-RAMP2 or -RAMP3 complexes being abolished. The effects were least prevalent for GRK2 and GRK3, which in some cases enhanced signalling. In general, the cAMP responses were more resistant to GRK overexpression than β-arrestin-recruitment. This is not surprising, as accumulation of cAMP is a result of signal amplification from the agonist-activated GPCR, while β-arrestin recruitment to the GPCR occurs at a 1:1 ratio. As a result, any loss of CLR-RAMP cell surface expression would be considered to have more of a deleterious effect upon β-arrestin recruitment than cAMP signalling. We therefore speculate that overexpression of GRKs might be leading to a reduction in CLR cell surface expression prior to agonist application.

To investigate this hypothesis, we investigated CLR cell surface expression in HEK293 cells using flow cytometry. CLR membrane expression was highest for CLR-RAMP1, and lowest for CLR-RAMP3, which showed only a small (∼1.5 fold) increase above the expression in the absence of RAMP. For all three CLR-RAMP complexes, we observed reductions in CLR cell surface expression when each GRK was overexpressed (Figure 5C, Supplementary Table S11). Consistent with the signalling data, CLR expression was reduced the least when GRK2 and GRK3 were overexpressed for each RAMP complex. We next wondered if the GRK-induced agonist-independent CLR internalisation was dependent upon β-arrestins. CLR cell surface expression was still reduced when GRKs were overexpressed in the absence of β-arrestins although there was not difference between the GRKs, suggesting the more detrimental effects of GRK4/5/6 are in part dependent upon β-arrestins (Figure 5D, Supplementary Table S11). Overall, these data confirm that CLR is able to undergo GRK-mediated internalisation in an agonist independent manner, via a mechanism which is largely independent of β-arrestins.

## Discussion

While G protein mediated signalling at CLR has been extensively studied for the three endogenous peptide agonists at each CLR-RAMP complex (Weston et al., 2016; Clark et al., 2021), studies investigating G protein independent events, such as β-arrestin recruitment and internalisation have only been investigated for specific CLR-RAMP-peptide combinations (Chang and Hsu, 2019; Hendrikse et al., 2020). Here we provide the first global characterisation of β-arrestin recruitment, internalisation and endosomal sorting of the three CLR-RAMP complexes when stimulated with CGRP, AM and AM2. Our results for the β-arrestin recruitment to CLR-RAMP1 and -RAMP2 when stimulated with their cognate ligands are consistent with previous studies in terms of potency, despite the use of different cell backgrounds and CLR constructs (Chang and Hsu, 2019; Hendrikse et al., 2020). Furthermore, we suggest that CLR undergoes internalisation by context-dependent mechanisms, with our results providing important implications for other class B1 GPCRs. Finally, we highlight that GRK overexpression is deleterious to both G protein-dependent and independent signalling and thus caution must be applied when GRKs are routinely expressed to increase β-arrestin recruitment.

**FIGURE 6:**
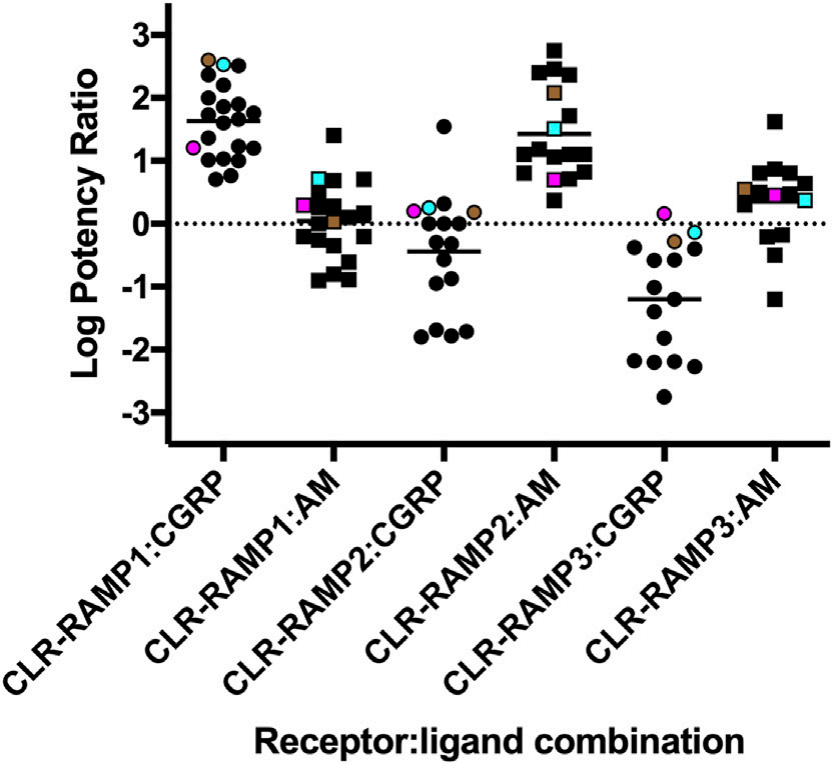
Agonist potency rations for CGRP, AM and AM2 at the three different CLR-RAMP complexes. The log Potency ratios (as determined from cAMP accumulation assays) are defined as log(EC50 AM2/EC50 agonist). Data compiled from Weston et al., (2016), Garelja et al., (2020), Clark et al., (2021) and Harris et al., (2021). HEK293T cells expressing CLR-Nluc are shown in *cyan*, HEK293 expressing CLR-Nluc are shown as *magenta* and HEK293T cells expressing HA-CLR are shown in *brown*.

Initially, we determined that the rank order of potency for the CGRP-based peptides at each CLR-RAMP complex was consistent across the different assays e.g., the most potent peptide at cAMP was the most potent at β-arrestin recruitment and internalisation. Whilst all peptides were able to elicit maximal responses in the cAMP accumulation assay (with their relative potencies largely agreeing with previous observations (Figure 6), this was not the case for β-arrestin recruitment or receptor internalisation. Across all combinations, CLR-RAMP1 seemed the most capable of recruiting β-arrestins and internalising, followed by CLR-RAMP2 (∼25%) and CLR-RAMP3 (∼15%). The 1:1 nature of β-arrestin recruitment and internalisation means that the cell surface expression level of the different CLR-RAMP complexes may largely explain this observed difference between RAMPs; CLR-RAMP2, and -RAMP3 expression was 52 and 28% of CLR-RAMP1 respectively. When looking at the RAMP instead, each displayed comparable surface expression, which then correlated with comparable levels of internalisation. Furthermore, it is likely that all three agonists reached a maximal level of cAMP accumulation due to the substantial amplification in the pathway, indicating a receptor reserve. Correspondingly, only CLR-RAMP1 displayed substantial detectable subcellular trafficking, in agreement with previous studies (Yarwood et al., 2017), with the other complexes only displaying small colocalisation with each of the endosomal markers. It was found for CLR-RAMP1 and CLR-RAMP2 that there was an increase in Rab7 and Rab11 colocalisation after receptor internalisation, suggesting that both slow recycling and degradative pathways are employed. Little colocalization with these endosomal markers was observed for CLR-RAMP3, presumably due to its lack of significant internalisation.

The receptor internalisation was confirmed to be β-arrestin dependent through the use of a cell line where β-arrestin1 and 2 were genetically KO. However, inhibition of the β-arrestin-AP2 interaction, using barbadin, had no effect on receptor internalisation. Furthermore, it was determined that whilst barbadin was able to significantly reduce cAMP accumulation, a major part of its action was independent of the β-arrestin. This is supported by the observation that the effects of barbadin were similar in β-arrestin KO HEK293 cells and were agonist dependent, with no effect on CGRP at CLR-RAMP1, which undergoes the greatest internalisation. cAMP signalling was enhanced by the removal of β-arrestins (and therefore loss of internalisation), and correspondingly decreased by their overexpression, suggesting β-arrestin recruitment and internalisation is utilised by CLR as a traditional desensitisation pathway, as observed for many Class A GPCRs. This agrees with previous findings that reducing internalisation of CLR-RAMP2 through C terminal tail deletions increases cAMP accumulation by the receptor (Kuwasako et al., 2010).

The final part of this study has considered the effects of increasing GRK expression on CLR β-arrestin recruitment and cAMP accumulation. Here, we have shown that over-expression of GRKs had detrimental effects on receptor signalling. In particular, GRK5 and 6 significantly impaired β-arrestin recruitment and ablated the majority of the cAMP response. This was found to be agonist-independent, with reduced surface expression of CLR observed when GRKs were overexpressed, as had been previously observed for CLR-RAMP2 (Kuwasako et al., 2016). Interestingly, there was still a reduction in CLR surface expression in the HEK293Δβ-arrestin1/2 cells indicating β-arrestin independency. Some GPCRs have been shown to use endophilin to mediate dynamin-dependent internalisation that is independent of AP2, β-arrestin and clathrin (Boucrot and Ferreira, 2017). This mechanism is unlikely for CLR since it is mediated via a proline rich motif in the intracellular loop 3, which is absent from CLR. Other Class B1 GPCRs are known to internalise via caveolae (Thompson and Kanamarlapudi, 2015; Fletcher et al., 2018), and it has been noted that GRKs are able to interact with caveolin-1, indicating a potential role in caveolae-mediated endocytosis (Carman et al., 1999). Furthermore, CLR has been shown to co-immunoprecipitate with caveolin-1, with stimulation with CGRP reducing membrane localisation of caveolin-1 (Tang et al., 2013). Our analysis of the C-terminal tail identifies a potential motif (I ^394/8.53b^LRRNWNQY ^402^) which conforms to one of the consensus caveolin-1 interacting domains (φXXXXφXXφ motif where X = any amino acid and φ = hydrophobic amino acids) (Couet et al., 1997). Thus, it is possible that the GRK-mediated agonist-independent internalisation of CLR could occur through caveolae. There is further precedent for context dependent mechanisms of internalisation as exemplified by the CB_1_ cannabinoid receptor where agonist-induced internalisation is β-arrestin mediated, but agonist-independent internalisation is clathrin-dependent, but β-arrestin-independent (Gyombolai et al., 2013).

These studies have significance for other investigations into GPCR β-arrestin recruitment/internalisation. If the magnitude of β-arrestin recruitment is weak to the GPCR of choice, addition of GRKs is often used to increase the signal (Mackie et al., 2019; Harris et al., 2021). As demonstrated for CLR, this is not always appropriate as it can lead to the opposite effects if agonist-independent phosphorylation of GPCRs occurs.

The data we have obtained related to agonist-dependent internalisation of CLR in the presence of barbadin (Figure 3C) appear contradictory when compared to our results obtained with the HEK293∆β-arr1/2 cell lines (Figure 4A). Barbadin has been suggested to block the interaction between β-arrestin and AP2 thereby inhibiting clathrin-dependent internalisation (Beautrait et al., 2017). It therefore seems unusual that barbadin did not block CLR internalisation. Barbadin has been shown to successfully block 5-HT2CR internalisation (He et al., 2021); β2-adrenergic (β2AR), V2-vasopressin (V2R), angiotensin-II type-1 (AT1R) receptors (Beautrait et al., 2017), free fatty acid receptor 2 (FFAR2) (Wang et al., 2020), protease-activated receptor 2 (PAR2) (Jung et al., 2021) and GLP-1R in our hands (Figure 3A). In the present study, we have used barbadin at the same concentrations as described previously and therefore we are unsure why it does not block agonist dependent CLR endocytosis. However, our observations that barbadin significantly attenuated cAMP signalling might provide some explanation. It is plausible that barbadin forces the β-arrestins to adopt a closed conformation on the agonist-occupied CLR which results in the G protein being unable to access the receptor, thus preventing signalling. This closed complex may then use an AP2 independent mechanism for internalisation, e.g. via caveolae. The studies from Yarwood et al., suggest that CLR-RAMP1 can also form a megaplex with both the G protein and the β-arrestins present to enable signalling from endosomal compartments (Yarwood et al., 2017). Presumably this complex uses AP2 for internalisation. As such our data suggests that the mechanism of CLR-RAMP internalisation may depend upon the conformation the β-arrestins adopt on the activated CLR. Further analysis will be required to determine the precise nature of barbadin’s action, since it also displayed some activity in the HEK293Δβ-arrestin1/2 cells suggesting off target effects. Significantly, the differences observed between small molecular inhibitors and genetic manipulation highlighted here demonstrate the need for complimentary approaches to enable a more complete picture of the processes used for GPCR signal transduction.

Our study has highlighted the differences between the GRK subtypes in their ability to presumably phosphorylate non-agonist bound CLR. The effects, in the absence of agonist, were least detrimental to signalling with GRK2 and GRK3. This is probably not surprising since these two GRKs contain a pleckstrin homology (PH) domain which binds Gβγ domains (Pitcher et al., 1992; Koch et al., 1993). The interaction with Gβγ aids GRK2/3 recruitment to the plasma membrane. Thus, generally only active GPCRs will bring about recruitment of GRK2/3. The fact that GRK2/3 expression still results in a small attenuation to signalling may be explained by the endogenous expression of constitutively active GPCRs in the HEK293 cells used in this study. GRK4/5/6, on the other hand, do not contain the PH domain and associate with the plasma membrane through palmitoylation of C-terminal cysteine residues or through an amphipathic helix that interacts with the phospholipids found in the membrane (Gurevich et al., 2012; reviewed; Gurevich and Gurevich, 2019). As such, these GRKs have the potential to interact with and phosphorylate GPCRs independently of agonist binding. Prior to our study, GRK4 has been shown to constitutively phosphorylate the dopamine D1 receptor (Rankin et al., 2006), and both GRK5 and GRK6 have been reported to phosphorylate inactive GPCRs *in vitro* and *in vivo* (Tran et al., 2004; Baameur et al., 2010; Li et al., 2015). Our data directly aligns with these reports and appears to be the first example of agonist independent GRK phosphorylation for Class B1 GPCRs. Indeed, given these previously documented examples, it does seem unusual as to why only a limited set of inactive GPCRs are phosphorylated by GRK4/5/6.

It is important to highlight that when we investigated cell surface expression of CLR in the presence of the different RAMPs in our HEK293 and HEK293Δβ-arrestin1/2 cells, little cell surface expression above background was detected when RAMP3 was co-expressed with HA-CLR. Despite this low expression, it was sufficient to enable a full cAMP response to be detected upon agonist stimulation, which showed equivalent potency to other reports using equivalent HEK293 cells lines (Weston et al., 2016; Clark et al., 2021). However, the reduced expression of CLR with RAMP3, and to a lesser extent RAMP2 will almost certainly explain why it may have been hard to detect β-arrestin recruitment and internalisation, and why the overexpression of GRKs had such a dramatic effect on these two complexes. It will be interesting to determine if this low CLR expression in the presence of RAMP3 is observed in other cell lines and endogenous cells such as those found in the cardiovascular system.

To the best of our knowledge, the data described here is the first documented evidence of CLR-RAMP complexes undergoing agonist-independent internalisation. Early reports have demonstrated agonist-dependent internalisation for the CLR-RAMP1 complex (Kuwasako et al., 2000; Hilairet et al., 2001), but this was not observed in the absence of an agonist. Detailed reports related to AM1R and AM2R internalisation are rarer in the literature (Schönauer et al., 2015) and as such our study is the first comprehensive analysis of these events for all three CLR-RAMP complexes.

Overall, our study has highlighted that CLR internalisation is complex, being dependent on β-arrestins but apparently independent of AP2. Future work would need to investigate any potential AP2 interacting domain on CLR, or identify if RAMPs themselves are able to mediate internalisation in a β-arrestin dependent manner. Whilst C terminal phosphorylation by GRKs is important for receptor desensitisation, it appears the intense over expression, used to amplify β-arrestin recruitment, can result in agonist independent internalisation of the receptor, so caution must be exercised when overexpressing these proteins.

## Data Availability

The raw data supporting the conclusion of this article will be made available by the authors, without undue reservation.
