# Supplementary material for: Determining the Effects of Differential Expression of GRKs and β-arrestins on CLR-RAMP Agonist Bias
Source: Front Physiol. 2022 Mar 29;13:840763. doi: 10.3389/fphys.2022.840763 (PMC9001978; doi:10.3389/fphys.2022.840763)
Supplement: Supplementary file 1 [file DataSheet1.PDF]

## Supplementary Information for:

# Determining the effects of differential expression of GRKs and β-arrestins on CLR-RAMP agonist bias.

Abigail Pearce[1†], Theo Redfern-Nichols[1†], Matthew Harris[1], David R. Poyner[2], Mark Wigglesworth[3] and Graham Ladds [1]

*** Correspondence:** Graham Ladds: grl30@cam.ac.uk

Supplementary Figures 1 – 9

Supplementary Tables 1 – 11

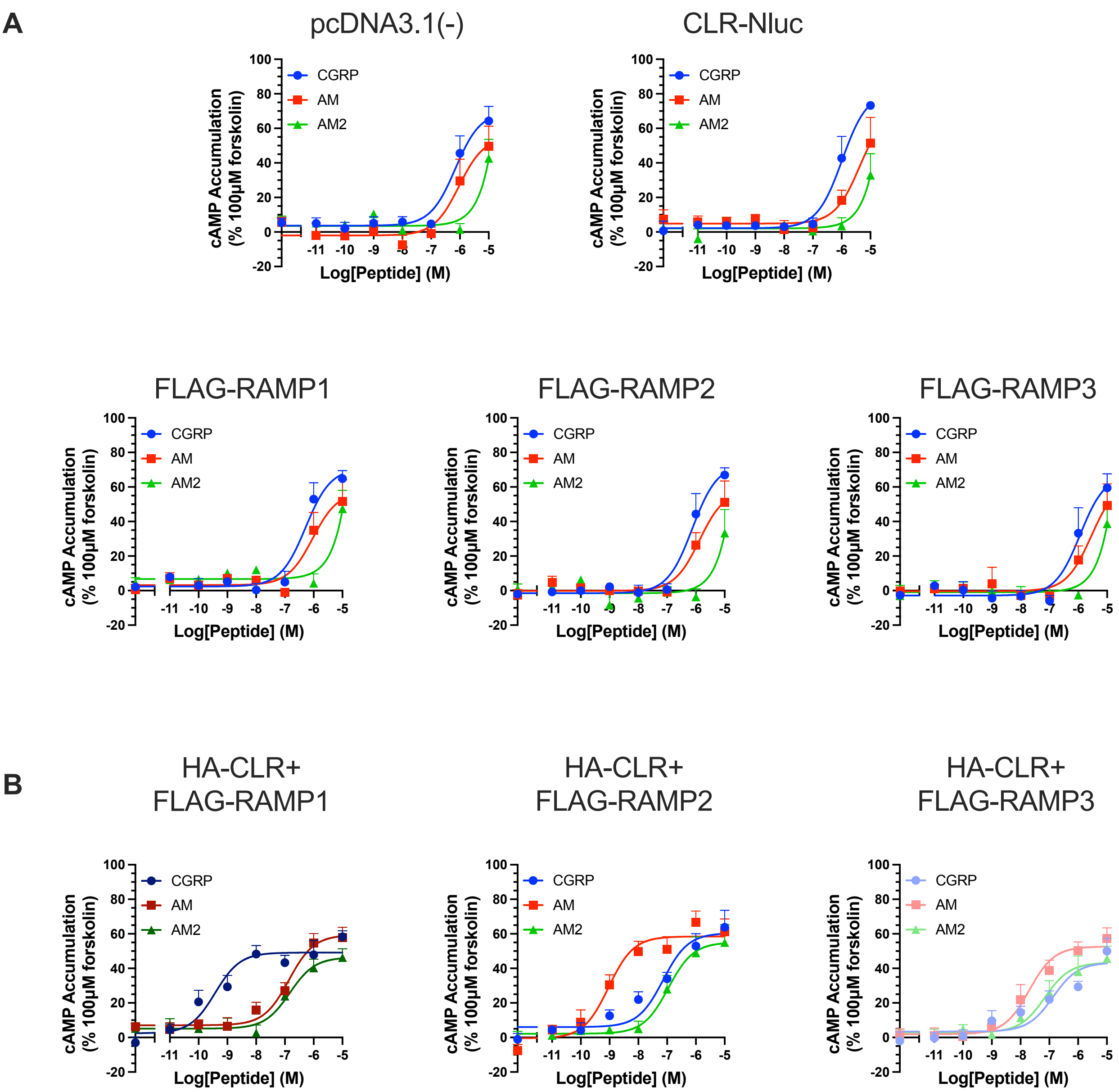


**Supplementary Figure 1. Quantification the cAMP accumulation from HEK293T cells transfected with various RAMP and CLR constructs.** **(A)** cAMP accumulation was determined in HEK293T cells using vectors alone (Mock), CLR-NLuc alone, and each individual RAMP construct alone following 30 min stimulation with CGRP, AM and AM2. (**B**) To assess the potency of the CLR-Nluc used in this study, HA-CLR was expressed in HEK293T cells and co-transfected with all three RAMPs and stimulated with the 3 agonists for 30 mins prior to quantification of cAMP accumulation. Data expressed to 100 µM forskolin and are the mean of *n* repeats with error bars indicating the SEM, where *n* ranges between 3 and 4 duplicates.

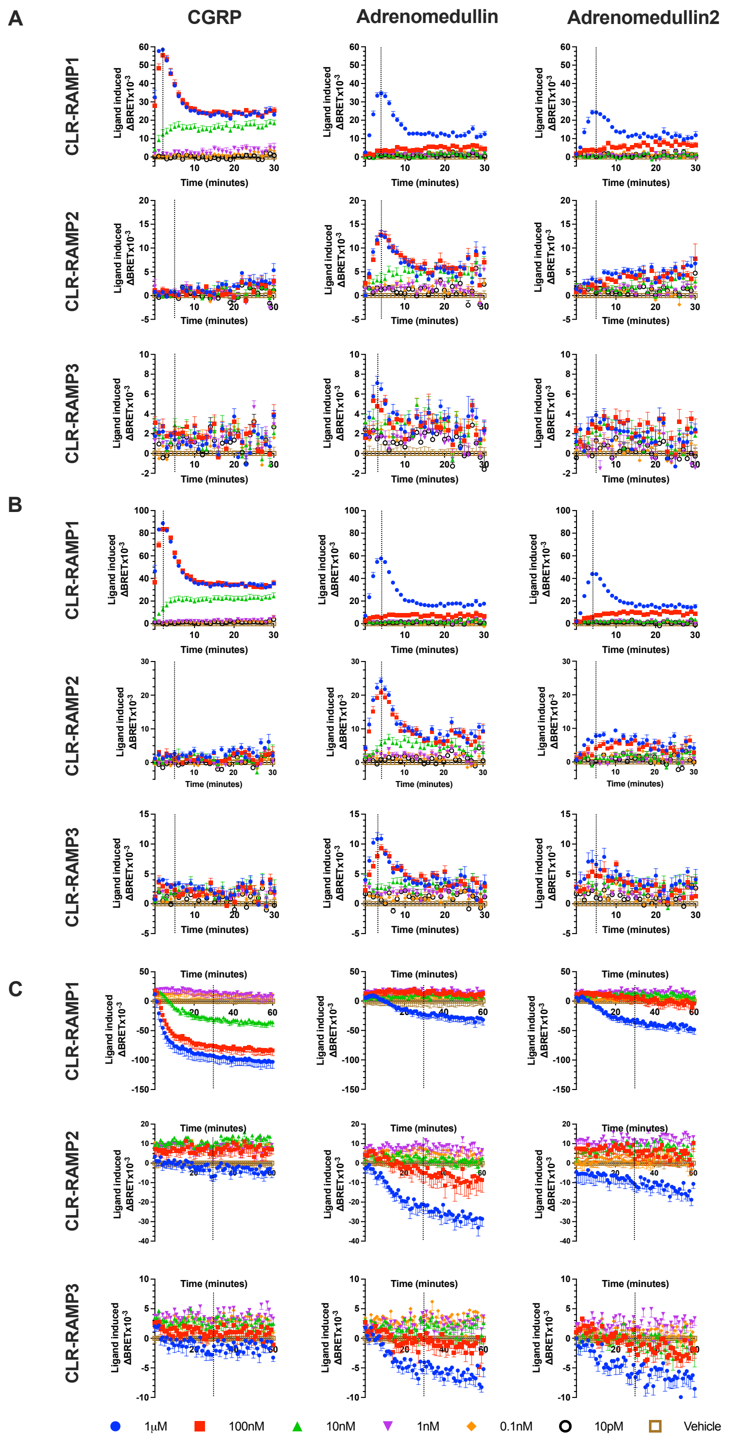


**Supplementary Figure 2**. **Quantification the effects of RAMP expression on CLR β-arrestin recruitment and receptor internalisation.** Ligand induced change in BRET ratio over time for the recruitment of β-arrestin1 (**A**), β-arrestin2 (**B**), and membrane dissociation (**C**) for CLR-RAMP1, CLR-RAMP2, and CLR-RAMP3 in response to CGRP, AM, and AM2, expressed in HEK293T cells. Dotted line indicates the time point used to generate concentration response curves. Data points represent the mean of *n* repeats with error bars indicating the SEM, where *n* ranges between 3 and 5 duplicates.

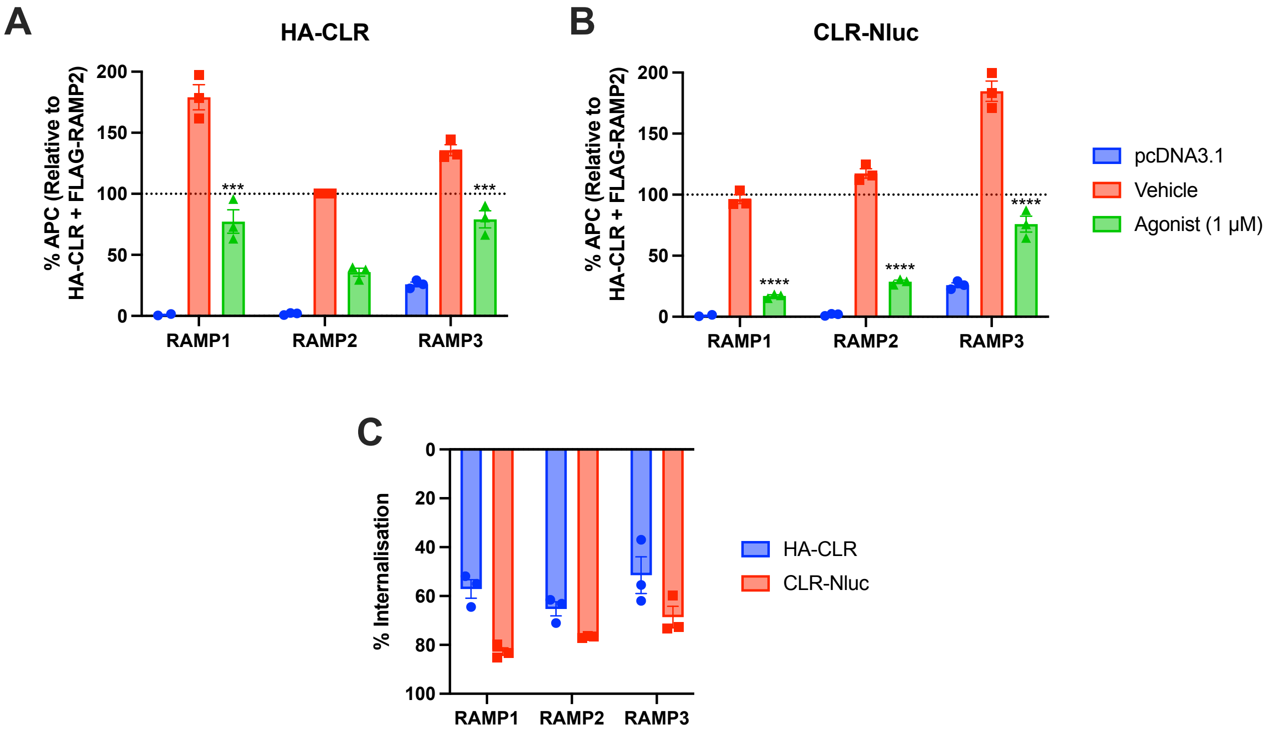


**Supplementary Figure 3. Surface expression and internalisation of FLAG-RAMPs in complex with HA-CLR or CLR-Nluc.** Surface expression of FLAG-RAMPs coexpressed with HA-CLR (A) or CLR-Nluc (B) following treatment for 30 minutes with vehicle or 1 μM CGRP (CLR-RAMP1), or AM (CLR-RAMP2/3). Data were assessed for statistical differences, at $p$<0.05, compared to vehicle treated cells using a one-way ANOVA with Dunnett's post-hoc test (***, $p$<0.001; ****, $p$<0.0001) or non-parametric Kruskal-Wallis test, as appropriate. (C) Percentage internalisation of FLAG-RAMPs when coexpressed with HA-CLR or CLR-Nluc.

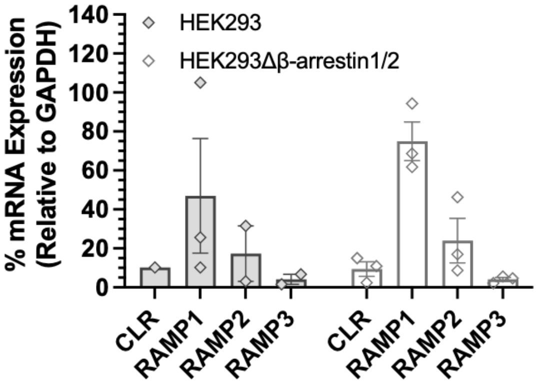


**Supplementary Figure 4**. **Expression of mRNA of CLR and RAMPs in HEK293 and HEK293Δβ-arrestin1/2 cells.** Expression was measured by RT-PCR in the cell lines indicated and standardised to GAPDH. Data points represent the mean of *n* repeats with error bars indicating the SEM, where *n*=3.

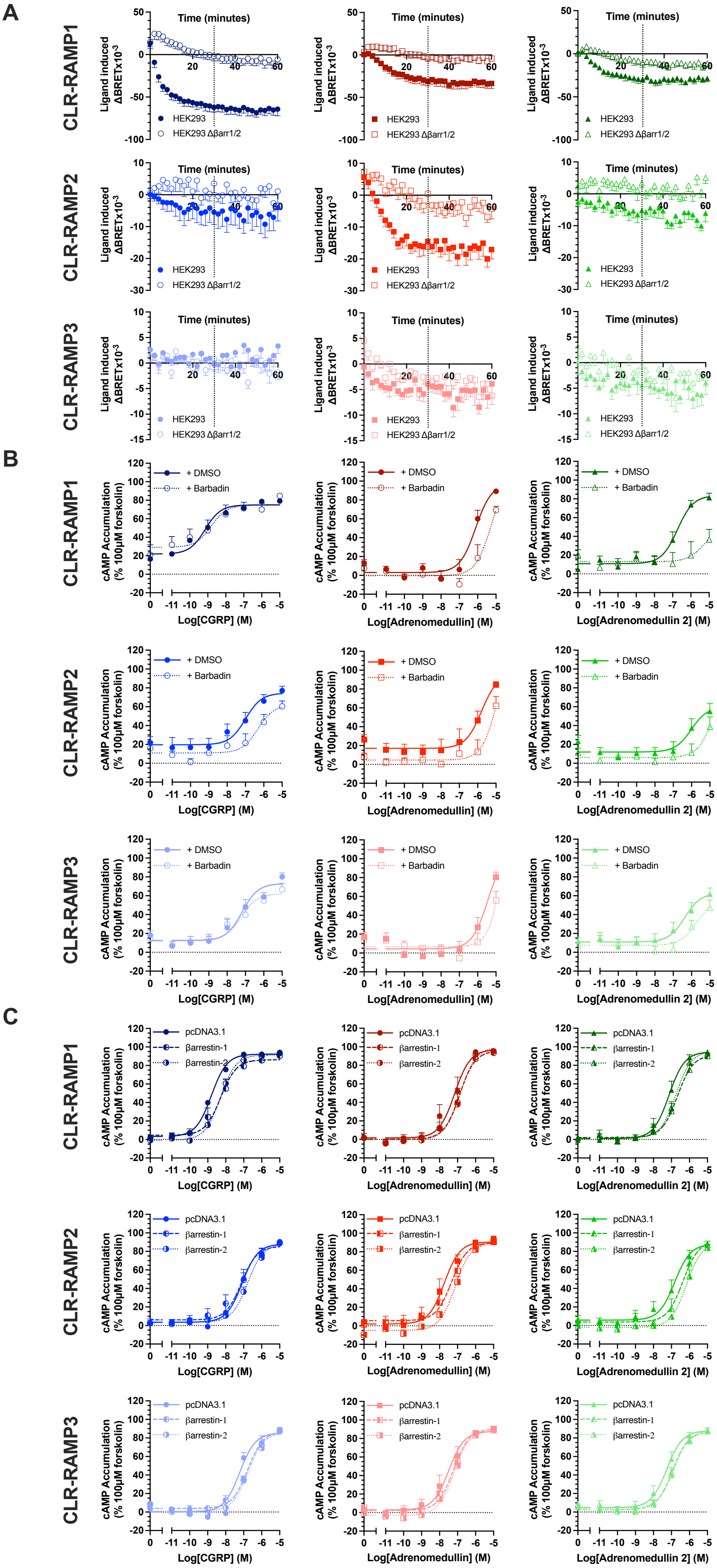

A
B
C
CLR-RAMP1
CLR-RAMP2
CLR-RAMP3
Time (minutes)
Ligand induced ΔBRETx10-3
HEK293
HEK293 Δβarr1/2
cAMP Accumulation (% 100μM forskolin)
+ DMSO
+ Barbadin
Log[CGRP] (M)
Log[Adrenomedullin] (M)
Log[Adrenomedullin 2] (M)
pcDNA3.1
βarrestin-1
βarrestin-2

**Supplementary Figure 5. Quantify effects of manipulation of β-arrestin expression upon CLR-RAMP internalisation and signalling.** (**A**) Ligand induced change in BRET ratio over time for the membrane dissociation of CLR-RAMP1, RAMP2, and RAMP3 in response to 1 μM peptide. Closed symbols indicate the response in HEK293 cells and open the response in HEK293Δβarr1/2 cells. (**B**) The effects of barbadin on cAMP accumulation in cells lacking β-arrestin1/2, looking at CLR-RAMP, RAMP2, and RAMP3. The response in DMSO treated cells is displayed at closed symbols, and open symbols indicate those pretreated with 100 μM barbadin. (**C**) Concentration response data for the effect of overexpressing β-arrestin1 (◐, dash line) or β-arrestin2 (◑, dotted line) on cAMP accumulation at CLR-RAMP1, RAMP2, or RAMP3. Data points represent the mean of *n* repeats with error bars indicating the SEM, where *n* ranges between 3 and 5 duplicates.

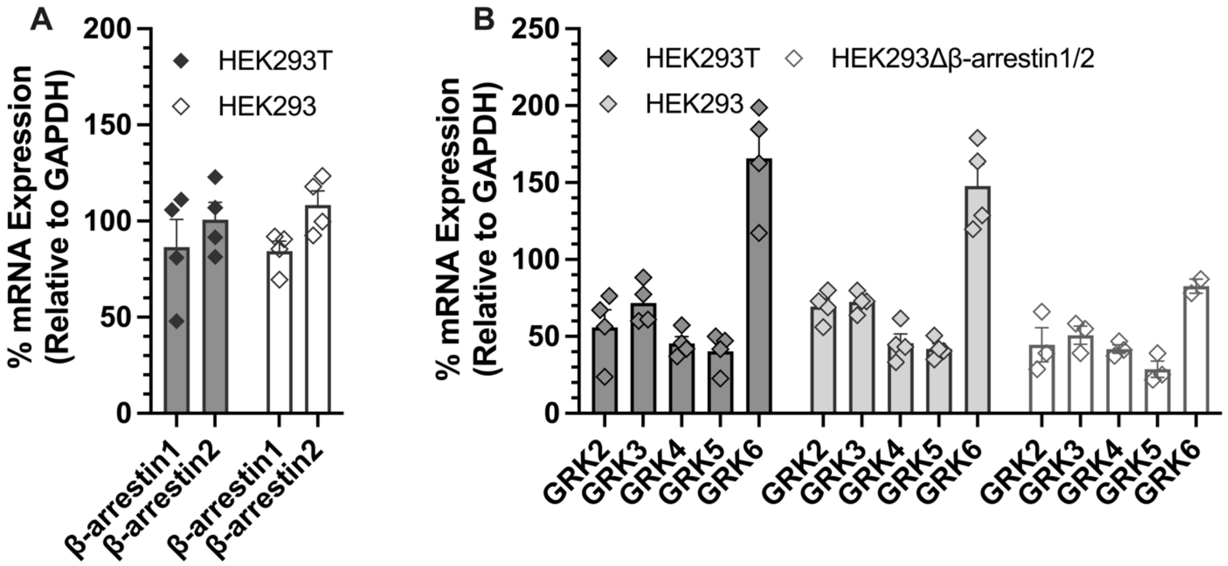


**Supplementary Figure 6. Expression of mRNA of β-arrestins and GRKs in different HEK293 cell lines.** Expression was measured by RT-PCR in the cell lines indicated and standardised to GAPDH. Data points represent the mean of *n* repeats with error bars indicating the SEM, where *n*=4.

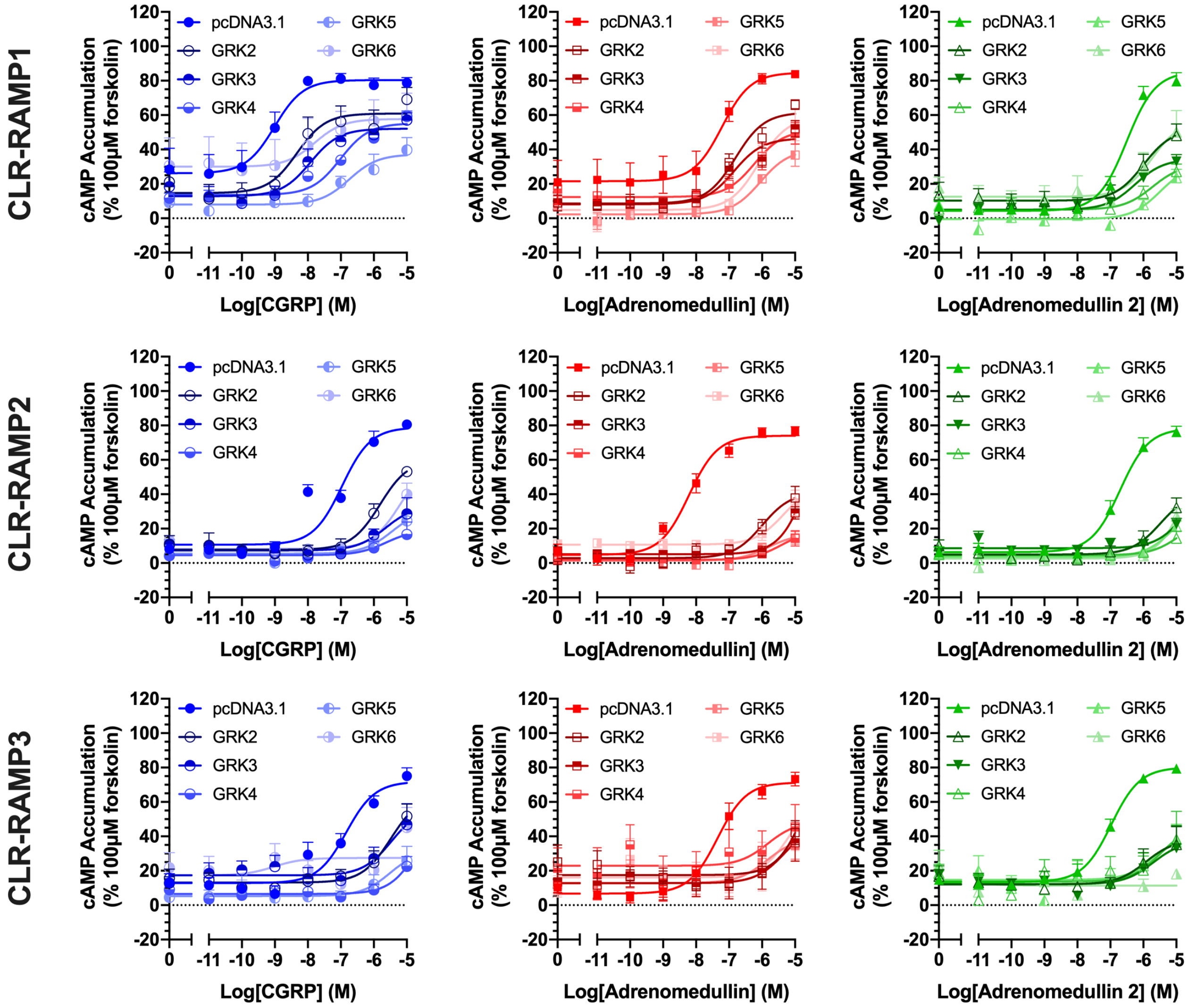


**Supplementary Figure 7. Effects of overexpressing GRK2-6 on CLR-RAMP cAMP accumulation.** HEK293T cell expressing CLR-RAMP complexes were stimulated with CGRP-based agonists for 30 mins in the presence of over expressed GRK2-6 and cAMP accumulation quantified. Data points represent the mean of *n* repeats with error bars indicating the SEM, where *n* ranges between 3 and 4 duplicates.

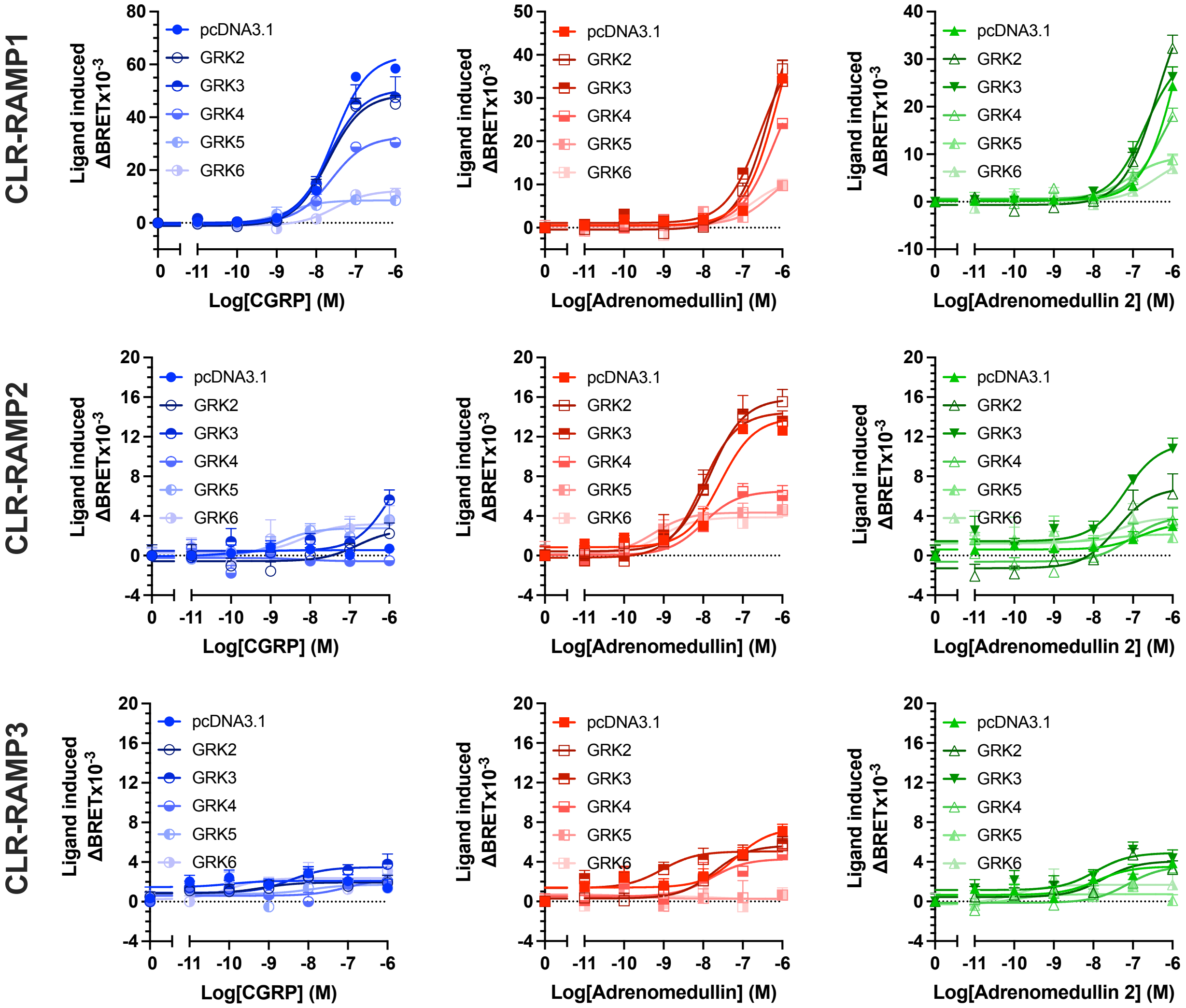


**Supplementary Figure 8. Effects of overexpressing GRK2-6 on CLR-RAMP β-arrestin1 recruitment.** HEK293T cell expressing CLR-RAMP complexes were stimulated with CGRP-based agonists in the presence of over expressed GRK2-6 and β-arrestin1 recruitment quantified. Data points represent the mean of *n* repeats with error bars indicating the SEM, where *n* ranges between 3 and 4 duplicates.

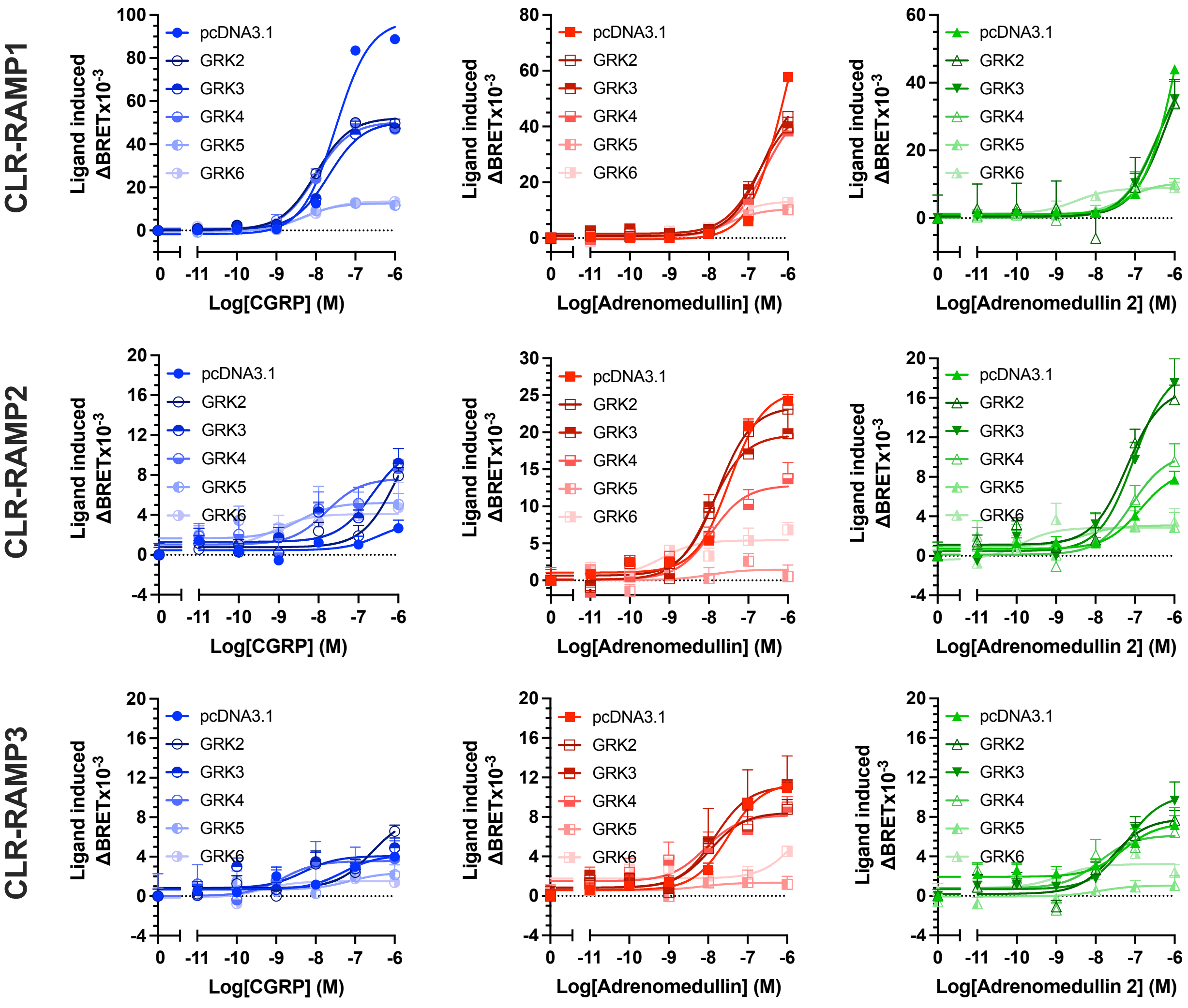


**Supplementary Figure 9. Effects of overexpressing GRK2-6 on CLR-RAMP β-arrestin2 recruitment.** HEK293T cell expressing CLR-RAMP complexes were stimulated with CGRP-based agonists in the presence of over expressed GRK2-6 and β-arrestin2 recruitment quantified. Data points represent the mean of *n* repeats with error bars indicating the SEM, where *n* ranges between 3 and 4 duplicates.

**Table S1.** $pEC_{50}$ and Emax values for the cAMP accumulation, β-arrestin1/2 recruitment, and internalisation of CGRP, AM, and AM2 at CLR-RAMP1 (upper), RAMP2 (middle), and RAMP3 (lower). N.R. designates cases where no response was recorded, or the response was too small to be able to accurately calculate potency and maximal values. Values are reported ± SEM, calculated from *n* repeats, where *n*=minimum of 3 repeats in duplicate.

| | CLR-RAMP1 | | | | | | | | |
|---|---|---|---|---|---|---|---|---|---|
| | CGRP | | | AM | | | AM2 | | |
| | $pEC_{50}$[a] | Emax[b] | n | $pEC_{50}$[a] | Emax[b] | n | $pEC_{50}$[a] | Emax[b] | n |
| **cAMP** | 9.04 ± 0.15 | 82.9 ± 3 | 3 | 7.21 ± 0.09 | 84.7 ± 3.0 | 3 | 6.5 ± 0.10 | 85.3 ± 3.7 | 4 |
| **β-arrestin1** | 7.56 ± 0.06 | 63 ± 2 | 3 | 6.04 ± 0.20 [#] | 63 ± 14 [#] | 3 | 5.79 ± 0.30 [#] | 63 ± 27 [#] | 3 |
| **β-arrestin2** | 7.49 ± 0.06 | 98 ± 3 | 3 | 6.13 ± 0.10 [#] | 98 ± 10 [#] | 3 | 5.90 ± 0.16 [#] | 98 ± 21 [#] | 3 |
| **Internalisation** | 7.78 ± 0.15 | -93 ± 7 | 4 | 6.18 ± 0.80 [#] | -44 ± 42 [#] | 3 | 6.23 ± 0.65 [#] | -63 ± 41 [#] | 3 |
| | **CLR-RAMP2** | | | | | | | | |
| | CGRP | | | AM | | | AM2 | | |
| | $pEC_{50}$[a] | Emax[b] | n | $pEC_{50}$[a] | Emax[b] | n | $pEC_{50}$[a] | Emax[b] | n |
| **cAMP** | 6.96 ± 0.15 | 78.9 ± 4.1 | 3 | 8.22 ± 0.11 | 74.1 ± 2.4 | 3 | 6.71 ± 0.10 | 78.3 ± 3.2 | 3 |
| **β-arrestin1** | N.R. | | 3 | 7.57 ± 0.17 | 14 ± 1 | 3 | 6.82 ± 0.66 | 3 ± 1 | 3 |
| **β-arrestin2** | N.R. | | 3 | 7.48 ± 0.09 | 26 ± 1 | 3 | 6.87 ± 0.19 | 9 ± 1 | 3 |
| **Internalisation** | 6.09 ± 1.36 [#] | -15 ± 32 [#] | 4 | 6.80 ± 0.30 [#] | -26 ± 6 [#] | 3 | 6.29 ± 0.82 [#] | -20 ± 18 [#] | 3 |
| | **CLR-RAMP3** | | | | | | | | |
| | CGRP | | | AM | | | AM2 | | |
| | $pEC_{50}$[a] | Emax[b] | n | $pEC_{50}$[a] | Emax[b] | n | $pEC_{50}$[a] | Emax[b] | n |
| **cAMP** | 6.84 ± 0.18 | 71.9 ± 4.7 | 4 | 7.35 ± 0.13 | 71.4 ± 3.2 | 4 | 6.97 ± 0.10 | 80.1 ± 2.7 | 4 |
| **β-arrestin1** | N.R. | | 3 | 7.04 ± 0.27 | 8 ± 1 | 3 | 7.72 ± 0.66 | 4 ± 1 | 3 |
| **β-arrestin2** | 7.31 ± 0.45 | 4 ± 1 | 3 | 7.49 ± 0.14 | 11 ± 1 | 3 | 7.31 ± 0.46 | 7 ± 1 | 3 |
| **Internalisation** | 7.25 ± 0.57 | -1 ± 1 | 5 | 6.92 ± 0.30 | -5 ± 1 | 4 | 6.77 ± 0.56 [#] | -8 ± 3 [#] | 4 |

[a] The negative logarithm of the agonist concentration required to produce a half-maximal response.

[b] The maximal response to the agonist expressed as percentage 100 µM forskolin for cAMP and as BRET units ($x10^{-3}$) for β-arrestin 1, β-arrestin 2 and internalization.

[c] Intrinsic relative activity as defined in the method.

[#] There is insufficient data to accurately fit a full logarithmic equation and such the values are a best estimate.

**Table S2.** Ligand induced change in BRET ratio between CLR-Nluc and RIT, Rab5a, Rab7, or Rab11-Venus, following 30-minute stimulation with CGRP, AM, or AM2. Values are the average of *n* repeats, where *n*=minimum of 3 repeats in duplicate, ± SEM. Significance calculated using Two-Way ANOVA, indicating the difference between Rab7 and Rab11 for the indicated RAMP-peptide combination with Tukey post-test (*, p < 0.05, **, p < 0.01, ***, p < 0.001, ****, p < 0.0001).

| | **CLR-RAMP1** | | | | | |
|---|---|---|---|---|---|---|
| | **CGRP** | | **AM** | | **AM2** | |
| | **ΔBRETx10$^{-3}$** | **n** | **ΔBRETx10$^{-3}$** | **n** | **ΔBRETx10$^{-3}$** | **n** |
| **RIT** | -83 ± 7 | 3 | -31 ± 8 | 3 | -44 ± 5 | 3 |
| **Rab5a** | -66 ± 5 | 3 | 3 ± 4 | 3 | -22 ± 4 | 3 |
| **Rab7** | 67 ± 2 **** | 3 | 34 ± 2 | 3 | 38 ± 2 ** | 3 |
| **Rab11** | 30 ± 5 **** | 3 | 17 ± 2 | 3 | 15 ± 4 ** | 3 |
| | **CLR-RAMP2** | | | | | |
| | **CGRP** | | **AM** | | **AM2** | |
| | **ΔBRETx10$^{-3}$** | **n** | **ΔBRETx10$^{-3}$** | **n** | **ΔBRETx10$^{-3}$** | **n** |
| **RIT** | -11 ± 3 | 3 | -26 ± 4 | 3 | -7 ± 2 | 3 |
| **Rab5a** | 1 ± 2 | 3 | -8 ± 2 | 3 | -1 ± 1 | 3 |
| **Rab7** | 8 ± 2 | 3 | 20 ± 3 | 3 | 6 ± 2 | 3 |
| **Rab11** | 8 ± 1 | 3 | 17 ± 3 | 3 | 6 ± 2 | 3 |
| | **CLR-RAMP3** | | | | | |
| | **CGRP** | | **AM** | | **AM2** | |
| | **ΔBRETx10$^{-3}$** | **n** | **ΔBRETx10$^{-3}$** | **n** | **ΔBRETx10$^{-3}$** | **n** |
| **RIT** | 0 ± 1 | 3 | -3 ± 1 | 3 | -2 ± 1 | 3 |
| **Rab5a** | -4 ± 1 | 3 | 1 ± 2 | 3 | 0 ± 1 | 3 |
| **Rab7** | 8 ± 1 | 3 | 13 ± 1 | 3 | 7 ± 1 | 3 |
| **Rab11** | 7 ± 1 | 3 | 10 ± 1 | 3 | 6 ± 1 | 3 |

**Table S3.** $pEC_{50}$ and Emax values for the cAMP accumulation of CLR-RAMP1, RAMP2, and RAMP3 in response to CGRP, AM, and AM2 when pre-treated with 1% DMSO (*v/v*) or 100 µM barbadin. Values calculated from *n* repeats, where *n*=minimum 3 repeats in duplicate. Significance calculated using Student's t-test and indicates difference between DMSO and barbadin treated cells (*, $p < 0.05$, **, $p < 0.01$, ***, $p < 0.001$, ****, $p < 0.0001$).

| | | **CGRP** | | | **AM** | | | **AM2** | | |
|---|---|---|---|---|---|---|---|---|---|---|
| | | **pEC50[a]** | **Emax[b]** | **n** | **pEC50[a]** | **Emax[b]** | **n** | **pEC50[a]** | **Emax[b]** | **n** |
| **CLR-RAMP1** | **DMSO** | 9.38 ± 0.48 | 81.5 ± 4.6 | 6 | 6.41 ± 0.27 | 88.0 ± 5.2 | 5 | 6.34 ± 0.13 | 79.8 ± 4.5 | 5 |
| | **Barbadin** | 9.13 ± 0.78 | 69.4 ± 6.0 | 6 | 5.58 ± 0.30 # | 91.4 ± 5.5 # | 5 | 4.93 ± 0.29 **# | 47.2 ± 8.9 *# | 5 |
| **CLR-RAMP2** | **DMSO** | 7.13 ± 0.59 | 93.7 ± 2.6 | 4 | 7.49 ± 0.36 | 80.3 ± 2.4 | 5 | 6.43 ± 0.27 | 78.1 ± 5.2 | 4 |
| | **Barbadin** | 6.51 ± 0.53 # | 97.1 ± 1.9 # | 4 | 5.33 ± 0.18 **# | 82.3 ± 14.3 # | 5 | 5.28 ± 0.33 *# | 23.5 ± 10.1 **# | 5 |
| **CLR-RAMP3** | **DMSO** | 6.84 ± 0.41 | 84.9 ± 2.7 | 6 | 6.32 ± 0.39 | 97.7 ± 1.5 | 6 | 6.40 ± 0.38 | 79.1 ± 7.1 | 5 |
| | **Barbadin** | 5.48 ± 0.20 *# | 78.9 ± 3.8 # | 6 | 4.86 ± 0.15 *# | 100.0 ± 0.0 # | 5 | 4.78 ± 0.13 **# | 19.7 ± 6.8 **# | 5 |

[a] The negative logarithm of the agonist concentration required to produce a half-maximal response.

[b] The maximal response to the agonist expressed as percentage 100µM forskolin for cAMP.

[#] There is insufficient data to perform a full logarithmic fit and such the values are a best estimate.

**Table S4.** Internalisation of CLR-RAMP1, RAMP2, and RAMP3 in response to 1 µM CGRP, AM, or AM2 in cells pre-treated with 1% DMSO (*v/v*) or 100 µM barbadin. Values are ligand induced change in BRET ratio between CLR-Nluc and RIT-Venus following 30 minute stimulation and are the average of *n* repeats, where *n*=minimum 3 repeats in duplicate. Significance calculated using Student's t-test and indicates difference between DMSO and barbadin treated cells $p < 0.05$.

| | | **CGRP** | | **AM** | | **AM2** | |
|---|---|---|---|---|---|---|---|
| | | **ΔBRETx10$^{-3}$** | **n** | **ΔBRETx10$^{-3}$** | **n** | **ΔBRETx10$^{-3}$** | **n** |
| **CLR-RAMP1** | **DMSO** | -83 ± 7 | 3 | -31 ± 8 | 3 | -44 ± 5 | 3 |
| | **Barbadin** | -83 ± 11 | 3 | -23 ± 6 | 3 | -49 ± 5 | 3 |
| **CLR-RAMP2** | **DMSO** | -11 ± 3 | 3 | -26 ± 4 | 3 | -7 ± 2 | 3 |
| | **Barbadin** | -13 ± 4 | 3 | -21 ± 4 | 3 | -4 ± 3 | 3 |
| **CLR-RAMP3** | **DMSO** | 0 ± 1 | 4 | -1 ± 2 | 4 | -1 ± 1 | 4 |
| | **Barbadin** | -1 ± 1 | 4 | 0 ± 1 | 4 | 0 ± 1 | 4 |

**Table S5.** Change in BRET ratio between CLR-Nluc and RIT-Venus when stimulated with 1μM CGRP, AM, or AM2 for 30 minutes. Values are the average of *n* repeats where *n*=minimum 3 repeats in duplicate. Significance is determined through One-Way ANOVA with Dunnett's post-test (*, $p < 0.05$, **, $p < 0.01$, ***, $p < 0.001$, ****, $p < 0.0001$).

| | | CGRP | | AM | | AM2 | |
|---|---|---|---|---|---|---|---|
| | | **BRET Ratiox10$^{-3}$** | **n** | **BRET Ratiox10$^{-3}$** | **n** | **BRET Ratiox10$^{-3}$** | **n** |
| **CLR-RAMP1** | **HEK293** | -62 ± 6 | 3 | -31 ± 5 | 3 | -30 ± 4 | 3 |
| | **Δβ-arr1/2** | -2 ± 5 **** | 3 | -3 ± 5 ** | 3 | -10 ± 5 * | 3 |
| **CLR-RAMP2** | **HEK293** | -8 ± 3 | 3 | -15 ± 3 | 3 | -5 ± 2 | 3 |
| | **Δβ-arr1/2** | 3 ± 1 * | 3 | 0 ± 3 *** | 3 | 3 ± 1 | 3 |
| **CLR-RAMP3** | **HEK293** | 0 ± 1 | 3 | -4 ± 1 | 3 | -4 ± 1 | 3 |
| | **Δβ-arr1/2** | 1 ± 1 | 4 | -3 ± 1 | 4 | -2 ± 1 | 3 |

**Table S6.** $pEC_{50}$ and Emax values for cAMP accumulation in HEK293 and HEK293Δβarr1/2 cells expressing CLR and RAMP1, 2, and 3 in turn. Values are reported ± SEM and are representative of *n* repeats where *n*=minimum of 3 repeats in duplicate. Significance is determined through Two-Way ANOVA with differences between HEK293 cells and HEK293Δβ-arr1/2 cells for the indicated RAMP-peptide combination with Tukey post-test (*, $p < 0.05$, **, $p < 0.01$, ***, $p < 0.001$, ****, $p < 0.0001$).

| | | **CGRP** | | | **AM** | | | **AM2** | | |
|---|---|---|---|---|---|---|---|---|---|---|
| | | **$pEC_{50}$[a]** | **Emax[b]** | **n** | **$pEC_{50}$[a]** | **Emax[b]** | **n** | **$pEC_{50}$[a]** | **Emax[b]** | **n** |
| **CLR-RAMP1** | **HEK293** | 7.39 ± 0.27 | 69.5 ± 5.7 | 4 | 6.48 ± 0.20 | 81.1 ± 7.9 | 4 | 6.18 ± 0.19 | 81.1 ± 8.1 | 4 |
| | **Δβ-arr1/2** | 7.78 ± 0.16 | 77.2 ± 4.2 | 4 | 7.04 ± 0.16 | 89.0 ± 5.4 | 4 | 6.57 ± 0.20 | 78.0 ± 6.5 | 4 |
| **CLR-RAMP2** | **HEK293** | 6.34 ± 0.20 | 81.4 ± 7.2 | 4 | 6.84 ± 0.13 | 83.8 ± 4.6 | 4 | 6.14 ± 0.17 | 79.3 ± 7.2 | 4 |
| | **Δβ-arr1/2** | 7.23 ± 0.19 | 80.3 ± 5.4 | 4 | 7.00 ± 0.13 | 80.5 ± 4.4 | 4 | 6.55 ± 0.22 | 78.3 ± 6.1 | 4 |
| **CLR-RAMP3** | **HEK293** | 6.63 ± 0.23 | 68.4 ± 6.8 | 4 | 6.92 ± 0.19 | 83.9 ± 6.8 | 4 | 6.47 ± 0.18 | 77.4 ± 6.8 | 4 |
| | **Δβ-arr1/2** | 7.09 ± 0.14 | 89.9 ± 4.8 | 4 | 6.90 ± 0.09 | 94.0 ± 3.6 | 4 | 6.62 ± 0.14 | 81.8 ± 4.4 | 4 |

[a] The negative logarithm of the agonist concentration required to produce a half-maximal response.

[b] The maximal response to the agonist expressed as percentage 100 μM forskolin for cAMP.

**Table S7.** $pEC_{50}$ and Emax values for cAMP accumulation in HEK293T cells expressing CLR and RAMP1, 2, and 3 in turn, with either β-arrestin1 or 2 overexpressed. Values are reported ± SEM and are representative of *n* repeats where *n*=minimum of 3 repeats in duplicate. Significance is determined through Two-Way ANOVA the difference between Vector and β-arr1/2 for the indicated RAMP-peptide combination with Tukey post-test (*, p < 0.05, **, p < 0.01, ***, p < 0.001, ****, p < 0.0001).

| | | CGRP | | | AM | | | AM2 | | |
|---|---|---|---|---|---|---|---|---|---|---|
| | | **pEC50[a]** | **Emax[b]** | **n** | **pEC50[a]** | **Emax[b]** | **n** | **pEC50[a]** | **Emax[b]** | **n** |
| **CLR-RAMP1** | **Vector** | 8.83 ± 0.25 | 94.2 ± 1.0 | 4 | 7.36 ± 0.31 | 100.6 ± 1.5 | 4 | 7.15 ± 0.18 | 96.5 ± 1.2 | 4 |
| | **β-arr1** | 8.40 ± 0.29 | 87.1 ± 2.8 | 4 | 6.97 ± 0.25 | 98.6 ± 2.4 | 4 | 6.69 ± 0.20 | 93.2 ± 3.5 | 4 |
| | **β-arr2** | 8.39 ± 0.17 | 91.4 ± 1.1 | 3 | 7.07 ± 0.24 | 99.3 ± 2.1 | 4 | 6.84 ± 0.14 | 95.0 ± 1.6 | 4 |
| **CLR-RAMP2** | **Vector** | 7.12 ± 0.23 | 89.2 ± 2.6 | 3 | 7.69 ± 0.23 | 93.8 ± 1.3 | 4 | 6.79 ± 0.26 | 92.4 ± 1.7 | 4 |
| | **β-arr1** | 7.12 ± 0.19 | 87.1 ± 3.2 | 4 | 7.38 ± 0.15 | 90.5 ± 1.9 | 4 | 6.41 ± 0.14 | 92.5 ± 3.6 | 4 |
| | **β-arr2** | 6.74 ± 0.13 | 89.9 ± 3.6 | 4 | 7.14 ± 0.12 | 90.7 ± 2.8 | 4 | 6.11 ± 0.10* | 91.9 ± 3.6 | 4 |
| **CLR-RAMP3** | **Vector** | 7.24 ± 0.09 | 85.2 ± 4.6 | 4 | 7.51 ± 0.24 | 90.7 ± 1.9 | 4 | 7.31 ± 0.16 | 88.3 ± 2.2 | 4 |
| | **β-arr1** | 6.81 ± 0.06 | 86.8 ± 4.4 | 4 | 7.27 ± 0.18 | 91.2 ± 2.0 | 4 | 6.89 ± 0.11 | 88.1 ± 2.2 | 4 |
| | **β-arr2** | 6.72 ± 0.10 | 86.6 ± 5.0 | 4 | 7.26 ± 0.16 | 89.3 ± 2.6 | 4 | 6.87 ± 0.08 | 88.8 ± 2.5 | 4 |

[a] The negative logarithm of the agonist concentration required to produce a half-maximal response.
[b] The maximal response to the agonist expressed as percentage 100 μM forskolin for cAMP.

**Table S8**. cAMP accumulation of CLR-RAMP1 (upper), RAMP2 (middle), and RAMP3 (lower) in response to CGRP, AM, and AM2 when GRK2-6 are over expressed. Values are representative of n repeats, where n=minimum 3 repeats in duplicate. N.R. designates cases where no response was recorded, or the response was too small to be able to accurately calculate potency and maximal values. Significance determined through One-Way ANOVA with Dunnett's post-test (*, $p < 0.05$, **, $p < 0.01$, ***, $p < 0.001$, ****, $p < 0.0001$).

| | **CLR-RAMP1** | | | | | | | | |
|---|---|---|---|---|---|---|---|---|---|
| | **CGRP** | | | **AM** | | | **AM2** | | |
| | **pEC$_{50}$[a]** | **Emax[b]** | **n** | **pEC$_{50}$[a]** | **Emax[b]** | **n** | **pEC$_{50}$[a]** | **Emax[b]** | **n** |
| **Vector** | 9.04 ± 0.15 | 82.9 ± 3.0 | 3 | 7.21 ± 0.10 | 84.7 ± 3.0 | 4 | 6.50 ± 0.10 | 85.3 ± 3.7 | 4 |
| **GRK2** | 8.33 ± 0.35 | 60.9 ± 5.0 | 3 | 6.81 ± 0.16 | 61.6 ± 3.7 * | 3 | 6.00 ± 0.32 | 52.0 ± 7.7 | 3 |
| **GRK3** | 7.92 ± 0.26 | 52.1 ± 3.5 * | 3 | 6.85 ± 0.18 | 46.5 ± 2.9 ** | 3 | 6.25 ± 0.25 | 34.5 ± 3.7 ** | 3 |
| **GRK4** | 7.01 ± 0.18 * | 55.3 ± 3.1 * | 3 | 6.23 ± 0.26 * | 51.3 ± 5.3 ** | 3 | 5.73 ± 0.33 [#] | 31.3 ± 5.6 **[#] | 3 |
| **GRK5** | 6.75 ± 0.36 * | 37.2 ± 4.6 *** | 3 | 6.05 ± 0.21 * | 40.0 ± 4.8 *** | 3 | 5.50 ± 0.47 [#] | 31.4 ± 10.8 **[#] | 3 |
| **GRK6** | 7.80 ± 0.96 | 57.7 ± 9.1 * | 3 | 6.10 ± 0.25 * | 58.9 ± 7.4 * | 3 | 5.74 ± 0.46 | 57.2 ± 13.2 | 3 |
| | **CLR-RAMP2** | | | | | | | | |
| | **CGRP** | | | **AM** | | | **AM2** | | |
| | **pEC$_{50}$[a]** | **Emax[b]** | **n** | **pEC$_{50}$[a]** | **Emax[b]** | **n** | **pEC$_{50}$[a]** | **Emax[b]** | **n** |
| **Vector** | 6.96 ± 0.15 | 78.9 ± 4.1 | 3 | 8.22 ± 0.11 | 74.1 ± 2.4 | 3 | 6.71 ± 0.10 | 78.3 ± 3.2 | 3 |
| **GRK2** | 5.81 ± 0.23 [#] | 60.3 ± 7.8 [#] | 3 | 5.99 ± 0.25 * | 41.2 ± 5.6 | 3 | 5.48 ± 0.41 [#] | 41.2 ± 11.0 [#] | 3 |
| **GRK3** | 5.64 ± 0.54 [#] | 33.5 ± 8.7 **[#] | 3 | N.R. | | 3 | N.R. | | 3 |
| **GRK4** | 5.62 ± 0.68 [#] | 19.4 ± 6.8 ***[#] | 3 | 5.40 ± 0.57 **[#] | 19.3 ± 7.5**[#] | 3 | N.R. | | 3 |
| **GRK5** | 5.45 ± 0.38 [#] | 31.2 ± 7.3 **[#] | 3 | 5.82 ± 0.46 **[#] | 16.9 ± 4.4**[#] | 3 | 5.09 ± 0.58 **[#] | 35.7 ± 19.9 [#] | 3 |
| **GRK6** | N.R. | | 3 | 5.34 ± 0.56 **[#] | 46.2 ± 16.0 [#] | 3 | N.R. | | 3 |
| | **CLR-RAMP3** | | | | | | | | |
| | **CGRP** | | | **AM** | | | **AM2** | | |
| | **pEC$_{50}$[a]** | **Emax[b]** | **n** | **pEC$_{50}$[a]** | **Emax[b]** | **n** | **pEC$_{50}$[a]** | **Emax[b]** | **n** |
| **Vector** | 6.84 ± 0.18 | 71.9 ± 4.7 | 4 | 7.35 ± 0.13 | 71.4 ± 3.2 | 4 | 6.97 ± 0.10 | 80.1 ± 2.7 | 4 |
| **GRK2** | 5.49 ± 0.36 [#] | 64.2 ± 13.6 [#] | 4 | N.R. | | 4 | 5.93 ± 0.49 [#] | 38.1 ± 7.8 [#] | 3 |
| **GRK3** | 5.46 ± 0.71 [#] | 57.0 ± 20.5 [#] | 3 | N.R. | | 4 | 5.70 ± 0.75 [#] | 37.4 ± 12.4 [#] | 3 |
| **GRK4** | N.R.. | | 3 | N.R. | | 4 | 5.49 ± 1.03 [#] | 46.1 ± 24.3 [#] | 3 |
| **GRK5** | 5.50 ± 0.88 [#] | 32.4 ± 16.1*[#] | 3 | 5.86 ± 0.74 [#] | 39.1 ± 12.0 *[#] | 4 | 5.6 ± 0.96 [#] | 42.2 ± 17.9 [#] | 4 |
| **GRK6** | N.R. | | 3 | N.R. | | 4 | N.R. | | 3 |

[a] The negative logarithm of the agonist concentration required to produce a half-maximal response.

[b] The maximal response to the agonist expressed as percentage 100μM forskolin for cAMP.

[#] There is insufficient data to perform a full logarithmic fit and such the values are a best estimate.

**Table S9**. β-arrestin1 recruitment of CLR-RAMP1 (upper), RAMP2 (middle), and RAMP3 (lower) in response to CGRP, AM, and AM2 when GRK2-6 are over expressed. Values are representative of *n* repeats, where *n*=minimum 3 repeats in duplicate. N.R. designates cases where no response was recorded, or the response was too small to be able to accurately calculate potency and maximal values. Significance determined through One-Way ANOVA with Dunnett's post-test (*, $p < 0.05$, **, $p < 0.01$, ***, $p < 0.001$, ****, $p < 0.0001$).

| | **CLR-RAMP1** | | | | | | | | |
|---|---|---|---|---|---|---|---|---|---|
| | **CGRP** | | | **AM** | | | **AM2** | | |
| | **pEC$_{50}$[a]** | **Emax[b]** | **n** | **pEC$_{50}$[a]** | **Emax[b]** | **n** | **pEC$_{50}$[a]** | **Emax[b]** | **n** |
| **Vector** | 7.56 ± 0.06 | 63 ± 2 | 3 | 6.04 ± 0.19 # | 63 ± 14 # | 3 | 5.78 ± 0.29 # | 63 ± 26 # | 3 |
| **GRK2** | 7.69 ± 0.10 | 49 ± 2 ** | 3 | 6.26 ± 0.13 # | 57 ± 7 # | 3 | 6.38 ± 0.14 # | 46 ± 5 # | 3 |
| **GRK3** | 7.69 ± 0.18 | 51 ± 4 ** | 3 | 6.56 ± 0.17 # | 43 ± 5 # | 3 | 6.69 ± 0.12 # | 32 ± 2 # | 3 |
| **GRK4** | 7.62 ± 0.09 | 33 ± 1**** | 3 | 6.19 ± 0.18 # | 39 ± 7 # | 3 | 6.23 ± 0.27 # | 28 ± 7 # | 3 |
| **GRK5** | N.R. | | 3 | 6.29 ± 0.55 # | 14 ± 7 **# | 3 | N.R. | | 3 |
| **GRK6** | 7.54 ± 0.22 | 12 ± 1 **** | 3 | 6.77 ± 0.31 # | 11 ± 2 **# | 3 | 6.42 ± 0.79 # | 10 ± 6 *# | 3 |
| | **CLR-RAMP2** | | | | | | | | |
| | **CGRP** | | | **AM** | | | **AM2** | | |
| | **pEC$_{50}$[a]** | **Emax[b]** | **n** | **pEC$_{50}$[a]** | **Emax[b]** | **n** | **pEC$_{50}$[a]** | **Emax[b]** | **n** |
| **Vector** | N.R. | | 3 | 7.57 ± 0.17 | 14 ± 1 | 3 | 6.82 ± 0.66 | 3 ± 1 | 3 |
| **GRK2** | 6.81 ± 0.74 | 3 ± 1 | 3 | 7.83 ± 0.14 | 16 ± 1 | 3 | 7.40 ± 0.37 | 7 ± 1 | 3 |
| **GRK3** | 5.97 ± 0.98 # | 11 ± 13 # | 3 | 8.02 ± 0.22 | 14 ± 1 | 3 | 7.20 ± 0.22 | 11 ± 1 ** | 3 |
| **GRK4** | N.R. | | 3 | 8.00 ± 0.26 | 7 ± 1 ** | 3 | 7.08 ± 0.49 | 4 ± 1 | 3 |
| **GRK5** | N.R. | | 3 | N.R. | | 3 | N.R. | | 3 |
| **GRK6** | N.R. | | 4 | N.R. | | 4 | N.R. | | 4 |
| | **CLR-RAMP3** | | | | | | | | |
| | **CGRP** | | | **AM** | | | **AM2** | | |
| | **pEC$_{50}$[a]** | **Emax[b]** | **n** | **pEC$_{50}$[a]** | **Emax[b]** | **n** | **pEC$_{50}$[a]** | **Emax[b]** | **n** |
| **Vector** | N.R. | | 3 | 7.17 ± 0.26 | 7 ± 1 | 3 | 7.91 ± 0.52 | 4 ± 1 | 3 |
| **GRK2** | N.R. | | 3 | 7.73 ± 0.25 | 6 ± 1 | 3 | 7.76 ± 0.51 | 4 ± 1 | 3 |
| **GRK3** | 8.44 ± 0.56 | 3 ± 1 | 3 | 9.04 ± 0.48 | 5 ± 1 * | 3 | 7.94 ± 0.44 | 5 ± 1 | 3 |
| **GRK4** | N.R. | | 3 | 7.78 ± 0.66 | 4 ± 1 | 3 | 7.14 ± 0.41 | 4 ± 1 | 3 |
| **GRK5** | N.R. | | 3 | N.R. | | 3 | N.R. | | 3 |
| **GRK6** | N.R. | | 4 | N.R. | | 4 | N.R. | | 4 |

[a] The negative logarithm of the agonist concentration required to produce a half-maximal response.

[b] The maximal response to the agonist expressed as BRET units ($x10^{-3}$).

[#] There is insufficient data to perform a full logarithmic fit and such the values are a best estimate.

**Table S10**. β-arrestin2 recruitment of CLR-RAMP1 (upper), RAMP2 (middle), and RAMP3 (lower) in response to CGRP, AM, and AM2 when GRK2-6 are over expressed. Values are representative of *n* repeats, where *n*=minimum 3 repeats in duplicate. N.R. designates cases where no response was recorded, or the response was too small to be able to accurately calculate potency and maximal values. Significance determined through One-Way ANOVA with Dunnett's post-test (*, p < 0.05, **, p < 0.01, ***, p < 0.001, ****, p < 0.0001).

| | CLR-RAMP1 | | | | | | | | |
|---|---|---|---|---|---|---|---|---|---|
| | CGRP | | | AM | | | AM2 | | |
| | **pEC$_{50}$[a]** | **Emax[b]** | **n** | **pEC$_{50}$[a]** | **Emax[b]** | **n** | **pEC$_{50}$[a]** | **Emax[b]** | **n** |
| **Vector** | 7.49 ± 0.06 | 98 ± 3 | 3 | 6.13 ± 0.10 # | 98 ± 10 # | 3 | 5.90 ± 0.16 # | 98 ± 21 # | 3 |
| **GRK2** | 8.01 ± 0.06 | 52 ± 1 **** | 3 | 6.61 ± 0.10 # | 54 ± 4 ***# | 3 | 6.22 ± 0.95 # | 54 ± 47 # | 3 |
| **GRK3** | 7.68 ± 0.13 | 50 ± 3 **** | 3 | 6.72 ± 0.11 # | 47 ± 3 ****# | 3 | 6.41 ± 0.21 # | 49 ± 8 # | 3 |
| **GRK4** | 8.00 ± 0.09 | 50 ± 2 **** | 3 | 6.59 ± 0.08 # | 48 ± 3 ****# | 3 | 6.49 ± 0.13 # | 45 ± 4 # | 3 |
| **GRK5** | 8.34 ± 0.19 ** | 13 ± 1 **** | 3 | 7.53 ± 0.25 *** | 11 ± 1 **** | 3 | N.R. | | 3 |
| **GRK6** | 8.13 ± 0.21 * | 14 ± 1 **** | 3 | 7.53 ± 0.25 *** | 13 ± 1 **** | 3 | N.R. | | 3 |
| | **CLR-RAMP2** | | | | | | | | |
| | CGRP | | | AM | | | AM2 | | |
| | **pEC$_{50}$[a]** | **Emax[b]** | **n** | **pEC$_{50}$[a]** | **Emax[b]** | **n** | **pEC$_{50}$[a]** | **Emax[b]** | **n** |
| **Vector** | N.R. | | 3 | 7.48 ± 0.09 | 26 ± 1 | 3 | 6.87 ± 0.19 | 9 ± 1 | 3 |
| **GRK2** | 6.02 ± 0.65 # | 15 ± 10 # | 4 | 7.78 ± 0.10 | 23 ± 1 | 4 | 7.20 ± 0.16 | 17 ± 1 * | 4 |
| **GRK3** | 6.66 ± 0.38 # | 11 ± 2 # | 3 | 7.95 ± 0.24 | 20 ± 2 * | 3 | 7.03 ± 0.19 | 19 ± 2 ** | 3 |
| **GRK4** | 7.80 ± 0.43 | 8 ± 1 | 3 | 7.93 ± 0.24 | 13 ± 1 *** | 3 | 7.07 ± 0.38 | 10 ± 2 | 3 |
| **GRK5** | N.R. | | 3 | N.R. | | 3 | N.R. | | 3 |
| **GRK6** | N.R. | | 3 | N.R. | | 3 | N.R. | | 3 |
| | **CLR-RAMP3** | | | | | | | | |
| | CGRP | | | AM | | | AM2 | | |
| | **pEC$_{50}$[a]** | **Emax[b]** | **n** | **pEC$_{50}$[a]** | **Emax[b]** | **n** | **pEC$_{50}$[a]** | **Emax[b]** | **n** |
| **Vector** | 7.31 ± 0.45 | 4 ± 1 | 3 | 7.49 ± 0.14 | 11 ± 1 | 3 | 7.31 ± 0.46 | 7 ± 1 | 3 |
| **GRK2** | 6.58 ± 0.43 # | 8 ± 2 # | 3 | 7.96 ± 0.25 | 8 ± 1 | 3 | 7.58 ± 0.29 | 8 ± 1 | 3 |
| **GRK3** | 8.31 ± 0.65 | 4 ± 1 | 3 | 7.83 ± 0.44 | 11 ± 2 | 3 | 7.22 ± 0.27 | 10 ± 1 | 3 |
| **GRK4** | 8.84 ± 0.94 | 4 ± 1 | 3 | 8.07 ± 0.43 | 8 ± 1 | 3 | 7.92 ± 0.50 | 6 ± 1 | 3 |
| **GRK5** | N.R. | | 3 | N.R. | | 3 | N.R. | | 3 |
| **GRK6** | N.R. | | 3 | N.R. | | 3 | N.R. | | 3 |

[a] The negative logarithm of the agonist concentration required to produce a half-maximal response.

[b] The maximal response to the agonist expressed as BRET units (x$10^{-3}$).

# There is insufficient data to perform a full logarithmic fit and such the values are a best estimate.

**Table S11**. Surface expression of HA-CLR expressed with FLAG-RAMP1-3 in turn, when GRK2/3/4/5/6 are overexpressed, in HEK293 (upper) and HEK293Δβ-arrestin1/2 (lower) cells. Data are expressed as a percentage of HA-CLR expressed with FLAG-RAMP1. Values are mean ± SEM of *n* experiments where *n*= 3-4 repeats. Significance determined through One-Way ANOVA with Dunnett's post-test except for CLR-RAMP1 where a Kruskal-Wallis One-Way ANOVA test with Dunn's post-test was used (*, $p < 0.05$, **, $p < 0.01$, ***, $p < 0.001$, ****, $p < 0.0001$).

| | **HEK293** | | | | | | | |
|---|---|---|---|---|---|---|---|---|
| | **CLR-Alone** | | **CLR-RAMP1** | | **CLR-RAMP2** | | **CLR-RAMP3** | |
| | **Surface Expression** | **n** | **Surface Expression** | **n** | **Surface Expression** | **n** | **Surface Expression** | **n** |
| **Vector** | 16.8 ± 2.6 | 3 | 100.0 ± 0.0 | 3 | 52.5 ± 2.5 | 3 | 28.4 ± 1.4 | 3 |
| **GRK2** | 13.9 ± 3.3 | 3 | 70.8 ± 5.7 | 3 | 31.0 ± 4.1**** | 3 | 16.1 ± 1.1**** | 3 |
| **GRK3** | 16.2 ± 3.3 | 3 | 76.8 ± 8.4 | 3 | 30.5 ± 2.2**** | 3 | 16.7 ± 0.8**** | 3 |
| **GRK4** | 11.2 ± 0.6 | 3 | 36.3 ± 2.3** | 3 | 17.3 ± 0.7**** | 3 | 11.6 ± 0.8**** | 3 |
| **GRK5** | 8.2 ± 1.1 | 3 | 34.7 ± 1.6** | 3 | 14.1 ± 0.6**** | 3 | 8.7 ± 1.2*** | 3 |
| **GRK6** | 8.0 ± 1.1 | 3 | 44.9 ± 6.6* | 3 | 19.7 ± 1.2**** | 3 | 8.1 ± 1.3**** | 3 |
| | **HEK293Δβ-arrestin1/2** | | | | | | | |
| | **CLR-Alone** | | **CLR-RAMP1** | | **CLR-RAMP2** | | **CLR-RAMP3** | |
| | **Surface Expression** | **n** | **Surface Expression** | **n** | **Surface Expression** | **n** | **Surface Expression** | **n** |
| **Vector** | 26.0 ± 1.1 | 3 | 100.0 ± 0.0 | 3 | 67.2 ± 3.7 | 3 | 35.9 ± 5.3 | 3 |
| **GRK2** | 22.8 ± 4.0 | 3 | 66.7 ± 0.1 | 3 | 36.4 ± 2.9*** | 3 | 21.5 ± 2.6* | 3 |
| **GRK3** | 28.9 ± 3.7 | 3 | 75.6 ± 3.9 | 3 | 31.4 ± 3.9**** | 3 | 23.4 ± 2.1* | 3 |
| **GRK4** | 21.5 ± 0.5 | 3 | 52.3 ± 2.4* | 3 | 23.2 ± 3.4**** | 3 | 22.4 ± 2.2* | 3 |
| **GRK5** | 20.5 ± 2.8 | 3 | 56.4 ± 10.0 | 3 | 27.5 ± 2.5**** | 3 | 19.2 ± 1.5** | 3 |
| **GRK6** | 18.5 ± 2.7 | 3 | 59.6 ± 4.6 | 3 | 35.2 ± 3.1**** | 3 | 15.3 ± 1.2** | 3 |
